# A chromosome-level genome assembly of *Rhizopus stolonifer* associated with passion fruit flower rot

**DOI:** 10.3389/fmicb.2026.1757919

**Published:** 2026-04-13

**Authors:** Jiaman Sun, Ge Chen, Donald M. Gardiner, Sabrina Morrison, Xiaonan Zhang, Elizabeth A. B. Aitken, Liu Yang, Andrew Chen

**Affiliations:** 1 Guangdong Provincial Key Laboratory of Conservation and Precision Utilization of Characteristic Agricultural Resources in Mountainous Areas, School of Life Science, Jiaying University, Meizhou, Guangdong, China; 2 Guangxi Crop Genetic Improvement and Biotechnology Key Lab, Guangxi Academy of Agricultural Sciences, Nanning, Guangxi, China; 3 Queensland Alliance for Agriculture and Food Innovation, The University of Queensland, St Lucia, QLD, Australia; 4 School of Agriculture and Food Sustainability, The University of Queensland, Brisbane, QLD, Australia

**Keywords:** flower rot of passion fruit, fungal effectors, genome assembly, Hi-C, PacBio HiFi, pathogenicity, *Rhizopus stolonifer*, whole-genome phylogenetics

## Abstract

Flower rot of passion fruit, caused by *Rhizopus stolonifer*, is an emerging disease that threatens commercial cultivation by reducing yield and fruit quality. Here, we present a chromosome-level genome assembly of *R. stolonifer* isolate PRFJ02, generated using PacBio HiFi sequencing combined with Hi-C chromatin conformation capture. The assembled genome spans 48.2 Mb across 11 chromosomes, with 98.9% completeness, and encodes 11,737 protein-coding genes. Functional annotation revealed a diverse repertoire of genes involved in metabolism, signal transduction, and protein processing, reflecting the organism’s broad biological capacity. Comparative genomic analyses across related *Rhizopus* species identified a conserved core gene set alongside lineage-specific gene clusters, and showed that PRFJ02 exhibits substantial gene family contraction relative to other genomes, with comparatively limited gene family expansion. Comparative effector analysis revealed a limited core effector gene set and a predominance of genome-specific effectors. Only a small number of effectors were shared exclusively among plant-infecting strains, suggesting that differences in effector repertoires alone do not explain differences among plant-infecting isolates. Analysis of carbohydrate-active enzymes (CAZymes) showed that glycoside hydrolases were the dominant class in secreted enzyme repertoire in PRFJ02 and other Rhizopus genomes. In PRFJ02, several CAZyme families, including glycoside hydrolase family GH28 and polysaccharide lyase families PL1 and PL41, were enriched, highlighting enzymes associated with pectin degradation. Together, these results provide a high-quality genome resource for *R. stolonifer* and new insights into its genomic features and secreted enzyme repertoire. They also provide a basis for future studies on the biology and management of flower rot in passion fruit.

## 1 Introduction

*Passiflora edulis*, commonly known as passion fruit, is an economically important fruit crop with yellow passion fruit (*Passiflora edulis* f. *flavicarpa* Deg.) and purple passion fruit (*Passiflora edulis* f. *edulis*) being the two most commercially important types for passion fruit production (Santos-Jiménez et al., 2022). Native to South America, passion fruit has been widely introduced and is cultivated throughout tropical and subtropical regions, including parts of Asia, Africa, and Oceania. Worldwide production of passion fruit is approximately 1.5 million tonnes per year (Zhang et al., 2023). In recent years, the cultivation area of passion fruit has expanded significantly in China. Its annual production has approached ~600,000 tonnes, with major cultivation in southern provinces such as Guangxi, Yunnan, and Guangdong (Zhang et al., 2023). This initiative is driven by increasing consumer demand and governmental initiatives supporting tropical fruit development (Chen et al., 2023). However, its production faces several significant challenges, particularly its susceptibility to biotic stresses, including flower rot (Sun et al., 2024), stem rot caused by *Fusarium solani* (Wu et al., 2025), Phytophthora blight caused by *Phytophthora nicotianae* (Chen et al., 2025) and various viral infections (Santos-Jiménez et al., 2025; Choi, 2025). These diseases not only reduce yield and fruit quality but also threaten the long-term sustainability of cultivation in affected regions.

Flower rot of passion fruit, caused by *Rhizopus stolonifer*, is an emerging and economically significant disease, particularly affecting its reproductive organs (Sun et al., 2024). The disease primarily manifests during the flowering and fruit-setting stages, resulting in flower necrosis, premature abscission, and significant yield losses (Manicom et al., 2003). The development of flower rot is promoted by warm and humid environmental conditions, making it particularly prevalent in tropical and subtropical cultivation regions. The disease has also been recently reported on another fruit tree crop, Indian jujube in southern China (Yang et al., 2025). Flower rot has been reported to cause yield losses up to 60% under favourable environmental conditions, posing a serious threat to commercial passion fruit production (Sun et al., 2024). *R. stolonifer* is one of the main pathogens in postharvest storage of more than 100 kinds of fruit and can infect a wide variety of hosts including strawberry, tomato, grape, peach, sweet potato and other fruits and vegetables (Liu et al., 2024). Despite its growing prevalence and substantial economic burden, comprehensive insights into its etiology, epidemiology and the development of effective, integrated management approaches remain insufficient. Possible control strategies include an integrated management approach for *Rhizopus stolonifer*. The cornerstone of management is rigorous cultural control, including field sanitation to remove infected plant debris and improved canopy management through pruning to reduce humidity. These practices are further supported by careful drip irrigation to keep flowers dry.

The plant’s response to such biotic stresses often involves complex defense mechanisms, including the rapid production of reactive oxygen species and the induction of programmed cell death at the infection site to restrict pathogen spread. The genomic characterization of the pathogen enables targeted approaches, such as leveraging the CAZyme and effector profiles to guide the development of enzyme inhibitors and inform resistance breeding in passion fruit varieties, paving the way for more sustainable long-term solutions.

Long-read genome sequencing was performed to gain insights into the pathogenic mechanisms of *R. stolonifer* and to identify genes related to its reproduction, metabolism, and stress responses. To generate a high-quality, chromosome-level genome assembly, we employed PacBio high-fidelity (HiFi) sequencing in combination with Hi-C chromatin conformation capture technology. This genome resource will contribute towards understanding the pathogenicity and aiding the development of improved strategies to manage flower rot of passion fruit in the future.

## 2 Materials and methods

### 2.1 Sample collection and fungal isolate

Passion fruit samples showing symptoms of flower rot were collected from fields in Wuhua, Guangdong (23.81°N, 115.71°E) on yellow passion fruit plants at flowering. Under aseptic conditions, the diseased flower pieces were surfaced sterilized using 1% bleach, rinsed in sterile water, blotted dry on paper towels, and then placed on potato dextrose agar (PDA) medium at 25 °C for 2 days. Resultant mycelia were sub-cultured onto new PDA plates, after which monoconidial cultures were generated. Isolate PRFJ02, was identified as *Rhizopus stolonifer* by morphology and molecular analysis (Sun et al., 2024). Pathogenicity of isolate PRFJ02 was further confirmed on passion fruit flowers and leaves through inoculation assays (Sun et al., 2024).

### 2.2 DNA extraction and genome sequencing

A starter culture of the isolate PRFJ02 was placed on PDA and grown for 3 days at 25 °C. From the subsequent growth, five mycelia disks (~10 mm in diameter) were transferred to 250 mL Erlenmeyer flasks containing 200 mL potato dextrose broth and incubated on shaker for 3 days at 25 °C. The mycelia mass was then filtered through sterile cheesecloth and rinsed with sterile distilled water to remove any remaining media. The mycelia mass was used to extract genomic DNA using a MagAttract HMW DNA kit (Cat no. 67563, Qiagen, Hilden, Germany) according to manufacturer’s instructions. Quantification of total DNA was performed with the Quant-iT PicoGreen dsDNA Assay Kit (Invitrogen, Thermo Fisher Scientific). The isolate was sequenced on an Illumina NovaSeq 6000 platform (NGS) and a PacBio SMRT flow cell, respectively. Genomic library for paired-end (2 × 150 bp) sequencing on an Illumina sequencing platform was constructed by Personalbio Technology Co., Ltd. (Shanghai, China) using the TruSeq DNA Sample Prep Kit v2 (Cat no. FC-121-2001, Illumina, San Diego, CA, USA). The required library for PacBio SMRT sequencing was prepared using the PacBio Template Prep Kit 1.0 (Pacific Biosciences, Menlo Park, CA, USA) according to the manufacturer’s instructions. Briefly, genomic DNA was sheared to an average fragment size of 10–15 kb by g-TUBE. The fragmented DNA was then ligated with specific adapters and subjected to enzymatic digestion. Size selection for fragments 10–15 kb was performed using the BluePippin system (Sage Science, Beverly, MA, USA) to construct SMRTbell library. The purified libraries were subsequently assessed for quality and size distribution using the Agilent Bioanalyzer 2100 (Agilent Technologies, Santa Clara, CA, USA) prior to sequencing on the PacBio Revio platform. Based on a new draft genome assembly generated from NGS and PacBio sequencing data, Hi-C sequencing data was performed to cluster the draft genome sequences into chromosomal groups and to determine the order and orientation of sequences within each chromosome, thereby improving the assembly to the chromosome level. The genomic DNA library for Hi-C sequencing was generated with TruSeq DNA PCR-free prep kit (Illumina, San Diego, CA, USA) and sequenced for paired-end (2 × 150 bp) on the Illumina NovaSeq 6000 platform.

### 2.3 Genome assembly

Quality control of raw sequencing data was conducted using fastp (v0.20.0) (https://github.com/OpenGene/fastp) to trim adapters (Chen, 2025), remove low-quality reads, and filter out reads with high levels of ambiguous bases. In advance of genome assembly, short-insert libraries (insert size <1,500 bp) obtained through NGS sequencing were analyzed to estimate basic genome parameters, such as genome size, heterozygosity, and repeat content by using GenomeScope 2.0 (Ranallo-Benavidez et al., 2020). HiFi reads obtained from PacBio sequencing were initially assembled *de novo* using Hifiasm (v0.18.5) (Cheng et al., 2021). To elevate the assembly to the chromosome level, Hi-C sequencing data were processed with HiC-Pro (v3.1.0) (Servant et al., 2015), with the restriction enzyme site set to GATC (DpnII) and all other parameters kept at default values. Hi-C reads were mapped using Chromap (v0.1.3) (Zhang et al., 2021) to generate alignments, which were used to anchor and scaffold the draft genome assembly to the chromosome level. The T2T (telomere-to-telomere) genome assembly was polished using NextPolish (v1.4.0) with PacBio HiFi reads to enhance the accuracy and quality of the genome assembly. The completeness of the final chromosome-level assembly was initially assessed using QUAST (Mikheenko et al., 2018) and then BUSCO v5.4.5 (Benchmarking Universal Single-Copy Orthologs, http://busco.ezlab.org) based on the lineage specific BUSCO dataset from the division Mucoromycota (Manni et al., 2021). Telomere repeats of 5’-ACAACC-3′ were detected on each chromosome sequence using the telomere identifier ‘tidk’ (Brown et al., 2025).

### 2.4 Independent hi-C validation

To independently assess Hi-C scaffold quality, the contig assembly and Hi-C raw sequencing reads were through the hic-scaffolding-nf pipeline (https://github.com/WarrenLab/hic-scaffolding-nf), which integrates Chromap v0.3.2 (Zhang et al., 2021), YaHS v1.2.2 (Zhou et al., 2022), SAMtools v1.23 (Li et al., 2009) and JuicerTools v1.22.01 (Durand et al., 2016). Hi-C reads were aligned to contigs with Chromap and then scaffolded with YaHS using GATC as the restriction enzyme site. The aligned BAM file generated by Chromap was converted to BED format using BEDtools v2.25 (Quinlan and Hall, 2010) and the contig fasta file was then indexed using SAMtools before both were run through a second scaffolding tool, SALSA2 v2.3 (Ghurye et al., 2017; Ghurye et al., 2019) to create a second scaffolded assembly. The SALSA2 and YaHS scaffold assemblies were run through BUSCO (v 5.8.0, AUGUSTUS, Mucorales) (Simão et al., 2015) and QUAST v5.3 (Gurevich et al., 2013) analyses vis Galaxy (The Galaxy Community, 2024) to assess assembly quality. Dot plots comparing scaffolded assemblies were made in D-Genies (Cabanettes and Klopp, 2018) with Minimap2 v2.28 (Li, 2018).

### 2.5 Gene prediction

The assembly was then annotated for functional elements including repeats, non-coding RNAs (ncRNAs) and predicted protein-coding genes. The tandem repeat sequences in the genome were predicted and masked using Tandem Repeats Finder (TRF, v4.10.0) (Benson, 1999). RepeatModeler (v2.0.4) (Flynn et al., 2020) and RepeatMasker (v4.1.4, http://www.repeatmasker.org) (Chen, 2004) were then utilized to detect and generate a genome-wide profile of repetitive elements in PRFJ02. Repeat Dfam database v3 was used for *de novo* model searches. To annotate ncRNAs in the genome, tRNA genes were predicted using tRNAscan-SE (v2.0) (Lowe and Eddy, 1997), and rRNA genes were identified using Barrnap (v0.9) (https://github.com/tseemann/barrnap). Protein-coding genes were predicted using AUGUSTUS (v2.5.5) (Stanke and Morgenstern, 2005), GlimmerHMM (version 3.0.4) (Majoros et al., 2004), and GeneMark-ES (version 4.71) software (Ter-Hovhannisyan et al., 2008). Homology-based gene prediction was conducted using Exonerate (v2.2.0) software by aligning protein sequences from closely related species to the assembled genome (Slater and Birney, 2005). To obtain a high-confidence, non-redundant gene set, gene models derived from *de novo* prediction and homology-based approaches were integrated using EVidenceModeler (EVM, v2.0.0) (Haas et al., 2008).

### 2.6 Functional annotation

Functional annotation of protein-coding genes was carried out using multiple databases. Protein sequences were aligned to the NCBI non-redundant (nr) protein database (release 2017.10.10) (Sayers et al., 2025) using DIAMOND (v2.0.14) (Buchfink et al., 2015) with an E-value threshold of 1e-6, and the best hit was retained for functional assignment. Functional annotation of protein-coding genes based on gene orthology was performed using eggNOG-mapper (V4.5) (Huerta-Cepas et al., 2017). KEGG and COG annotations were obtained by mapping gene sequences to their respective databases (Kanehisa and Goto, 2000; Tatusov et al., 2000). To obtain more precise annotations, the Swiss-Prot database was queried using BLAST with default parameters (Bairoch and Apweiler, 2000). InterPro (v66.0, release 2017.11.23) was used to identify conserved protein domains and motifs (Finn et al., 2017). The resulting InterPro entries were processed in InterPro2GO database to obtain GO terms, which were then mapped to a list of GO slims using map2slim tool (https://github.com/elhumble/map2slim, accessed on 18 February 2025). Protein domains were identified using HMMER (v3.3.2) (Eddy, 2011) against the Pfam database (release 35.0) (Paysan-Lafosse et al., 2025). Functional annotation of protein-coding genes related to pathogen–host interactions was performed by aligning protein sequences against the PHI-base (Pathogen–Host Interactions database, version 4.17) (Urban et al., 2025), using BLASTP with an E-value cutoff of 1e-5 (Camacho et al., 2009). The best hit was retained for annotation. Searches in the CAZy database (http://www.cazy.org) (Cantarel et al., 2009) and the Database of Fungal Virulence Factors (DFVF) (http://sysbio.unl.edu/DFVF/index.php) (Lu et al., 2012) were performed using DIAMOND (v2.0.14) to classify carbohydrate-active enzymes and fungal virulence factors, respectively.

### 2.7 Subcellular localisation analysis

The signal peptide sequences in the predicted protein-coding genes were assessed using SignalP (v5.0) (Almagro Armenteros et al., 2019a) and TargetP (v2.0) (Almagro Armenteros et al., 2019b). The analysis classified the signal peptides into different secretory pathways, with the majority predicted to be secreted via the classical ER/Golgi pathway, while a smaller subset was predicted to contain targeting sequences to direct proteins to other cellular locations such as the mitochondria. The transmembrane helix structures of the predicted protein-coding genes were predicted using TMHMM (v2.0) (Krogh et al., 2001). Additionally, the prediction of effector proteins in pathogenic fungi was performed using EffectorP (v3.0) (Sperschneider et al., 2022), which identifies candidate effectors based on machine learning models trained on known effector sequences.

### 2.8 Comparative genomic analysis

#### 2.8.1 Assessment of *Rhizopus* genomes for comparative analysis

There are seven *R. stolonifer* genome assemblies currently available in the public domain (Supplementary Table S1). Out of these assembly, only four of them had a BUSCO completeness score of over 90%. These include our own (host/origin = passion fruit), B9770 (Contaminated product), BLUCD01 (Tomato) (Mesquida-Pesci et al., 2025), and gzRhiStol (Culture). The other three were not used due to low BUSCO scores (23.8 to 81.7%). Assemblies for each of *R. arrhizus* (GL38), *R. microsporus* (gzRhiMicr1), *R. delemar* (RO3), and *Mucor circinelloides* (CBS 3944.68, outgroup) were also selected based on a high BUSCO score of over 90% (Supplementary Table S1).

#### 2.8.2 Orthologous gene clustering

To obtain orthologous gene clusters specific to *R. stolonifer* isolates versus those shared among multiple species of this genus, gene-encoding protein clustering were performed using the OrthoMCL algorithm (Li et al., 2003) and an e-value threshold of 0.01 and an inflation value of 1.50 in the software OrthoVenn3 (https://orthovenn3.bioinfotoolkits.net, accessed on 27 August 2025) (Sun et al., 2023).

GO term enrichment was performed by comparing a selected set of specific gene clusters (unique or shared among genomes) with these of the background set (all single-copy gene clusters). A species phylogeny was inferred in OrthoVenn3 using Fast tree and a maximum-likelihood approach under the JTT + CAT amino-acid substitution model.

Expansion and contraction of orthogroups within *Rhizopus stolonifer* genomes was performed using CAFE5 (Mendes et al., 2021) within Orthovenn3. While genera under the subkingdom Mucoromyceta were estimated to have diverged earlier than 12 Mya (Zhao et al., 2023), divergence time between specific genera or within Rhizopus was not found on Timetree5 (https://timetree.org). Because Orthovenn3 only considers copy-number differences for expansion and contraction inference, the assumed divergence time does not influence the counts reported. The analysis therefore reflects observed gene family variation among *R. stolonifer* isolates rather than any time-scaled evolutionary rates.

#### 2.8.3 Genome-wide analysis of effector genes

Cytoplasmic and apoplastic effectors were initially predicted for all eight genomes using EffectorP v3.0, with integral membrane proteins removed using TMHMM v2.0, as described in Section 2.7. The predicted effectors were then divided into two sets. The apoplastic effector set included both apoplastic-only and dual-localised effectors with a dominant apoplastic signal, while the cytoplasmic effector set included cytoplasmic-only and dual-localised effectors with a dominant cytoplasmic signal.

For the cytoplasmic set, additional filtering criteria were applied using a custom script: protein length ≤300 amino acids, a prediction probability ≥0.8, and a minimum cysteine content of 3% relative to the total amino acid length of each gene.

Apoplastic or cytoplasmic candidate effector protein sequences from each genome were concatenated into a single file and then processed using a custom script to conform to the GenomeID|ProteinID structure.

MMseq2 (v 18-8cc5c) (Steinegger and Söding, 2017) was used to cluster homologous candidate effector protein sequences across the eight genomes. We ran MMseqs2 clustering on the protein sequences using a sequence identity threshold of 50% (--min-seq-id 0.5) and a coverage threshold of 80% (--cov-mode 0 with coverage ≥ 0.8`). These parameters ensured that sequences were grouped into clusters only if they were at least 50% identical across 80% of their length, balancing sensitivity and specificity. This allowed detection of both closely related homologs and more divergent sequences. The resulting clusters formed the basis for the presence/absence analyses and comparative genomics across the eight fungal genomes.

The cluster.tsv file containing cluster and genome-specific sequence IDs was then imported into R. Using the tidyr and dplyr R packages, we computed the dataset into a binary presence/absence matrix, where each row represented a cluster and each column represented a genome. Empty rows and columns were removed from the matrix. These contained only zeroes, which corresponded to clusters without any sequences. The cleaned binary matrix was saved as a data frame and then plotted using the pheatmap package. Clustering of both rows (clusters) and columns (genomes) was performed using binary distance metrics (Jaccard index) and average linkage (UPGMA). Presence and absence of clusters across each genome then allowed shared and unique effector candidate gene content to be visualised. Unique and shared gene content in the Rhizopus genomes were accessed for enriched GO terms using the R package clusterprofiler.

#### 2.8.4 CAZyme-enriched secretome analysis

Protein sequences from the eight genomes were searched against the dbCAN HMM database (v14) using HMMER (v3.4). The dbCAN software (v3.0.7) provided the CAZyme HMM profiles and helper scripts to facilitate the identification and annotation of carbohydrate-active enzymes in these genomes (Zhang et al., 2018). DIAMOND (v 0.9.19) was performed using the genome protein sequences as queries against the CAZy database (v 07262023, http://bcb.unl.edu/dbCAN2/) and a cutoff e-value of 1e-5. The hmm outputs were then filtered and only proteins with corresponding hits in the DIAMOND output were subsequently retained. Furthermore, integral membrane proteins were filtered from the eight genomes using TMHMM (v2.0), and only proteins predicted to carry a signal peptide by SignalP (v6.0) were subsequently retained. This set represents the secreted CAZyme repertoire of each genome.

A genome × HMM family matrix was constructed to characterise the distribution of secreted CAZyme families across the eight fungal genomes. In this matrix, each row represents an HMM family and each column represents a genome, with values corresponding to the number of signal peptide (SP)–containing proteins matching each HMM. Hierarchical clustering was performed on both axes to visualise similarities among genomes and HMM families, using Euclidean distance and complete linkage. HMM family counts were log-transformed using log10(count + 1) to reduce skewness and minimise bias towards highly abundant families.

Raw HMM counts were additionally used to identify families that were predominantly represented in the *Rhizopus stolonifer* PRFJ02 genome. Two criteria were applied. First, globally dominant families were defined as HMM families for which PRFJ02 contained the highest number of proteins among all eight genomes. Second, *R. stolonifer*-specific dominant families were defined as those for which PRFJ02 had the highest count among the four *R. stolonifer* genomes, regardless of whether another Rhizopus species had a higher count overall.

To further examine variation in secretome and CAZyme composition among genomes, a principal component analysis (PCA) was performed using the HMM family count matrix. Each row corresponded to an HMM family (e.g., AA, CBM, CE, GH families), and each column represented a genome. Raw counts were log-transformed using log10(count + 1) to stabilise variance across families with different abundance levels. HMM families showing zero variance across all genomes were removed prior to analysis to avoid computational issues. The matrix was then transposed so that genomes represented observations and HMM families represented variables. PCA was conducted using the prcomp function in R without additional scaling. The first principal component (PC1) captures the largest proportion of variation among genomes, while the second principal component (PC2) represents the second-largest independent source of variation.

### 2.9 Statistical analysis

The completeness of the final genome assembly was quantitatively assessed using BUSCO analysis, which provides a score based on the percentage of evolutionarily conserved, single-copy genes found in the assembly. A Fisher’s Exact Test was used to determine significant overrepresentation of GO terms, with a significance threshold of *p* < 0.01. Multiple testing correction was applied to control the false discovery rate.

## 3 Results and discussion

### 3.1 Pathogen isolation

**Figure 1:**
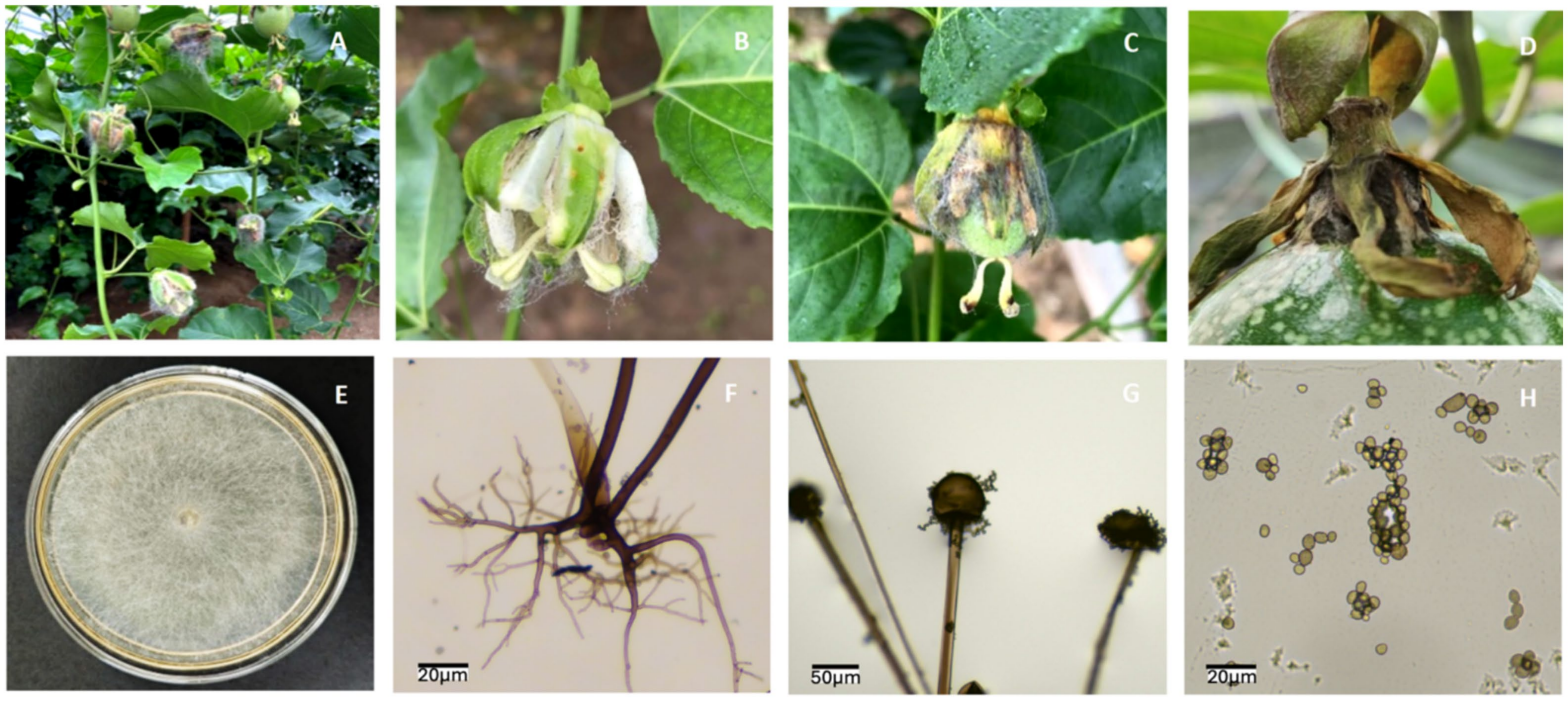
Symptoms of flower rot on passion fruit and morphological characteristics of *Rhizopus stolonifer*. **(A)** Symptomatic passion fruit plants infected with flower rot in Guangdong, China. (**B**,**C**) The mold layer covered the flowers and fruits, respectively. (**D**) The rotted pedicel abscised easily from the fruit. (**E)** *Rhizopus stolonifer* isolate PRFJ02 grown on potato dextrose agar medium. (**F**–**H**) Morphology of rhizoids, sporangia, and sporangiospores of *Rhizopus stolonifer* PRFJ02, respectively. Bars correspond to a scale of 20 μm in panel **(F,H)** and 50 μm in panel **(G)**.

Flower rot of passion fruit caused by *R. stolonifer* has led to significant losses to the local passion fruit industry, with severe field infections observed (Figure 1A). *R. stolonifer* isolate PRFJ02 was isolated from flowers in a local passion fruit plantation in Guangdong, which exhibited severe rot symptoms, such as a mold layer covering the floral tissues and fruits and subsequent abscission of the pedicel (Figures 1B–D). The fungal colonies on PDA exhibited diffuse, cottony mycelia that are hyaline and aseptate (Figure 1E). The rhizoids, sporangiophores, sporangia, and sporangiospores of *Rhizopus stolonifer* PRFJ02 were clearly observed and their morphology was visualised and confirmed under a microscope (Figures 1F–H). Sporangiophores were erect, light brown, typically measuring 0.56 (0.22–1.10) mm in length and 6.1 (3.18–10.87) μm in width at the base, and arose from stolons opposite the rhizoids. Sporangia were dark-coloured, nearly spherical, ranging from 23.45 to 92.85 μm in diameter. Upon maturation, the sporangial wall dissolved, releasing numerous dark, ovoid to subglobose sporangiospores with an average size of 3.56 μm × 2.82 μm (*n* = 100).

### 3.2 Genome assembly of PRFJ02

K-mer analysis using *k* = 19 in GenomeScope (v 1.0) was performed on short-insert (< 1,500 bp) PacBio libraries as input to predict a maximum genome size of 49 Mb, a genome repeat length of 28.4 Mb and a heterozygosity of 0.18% (Supplementary Figure S1).

**Figure 2:**
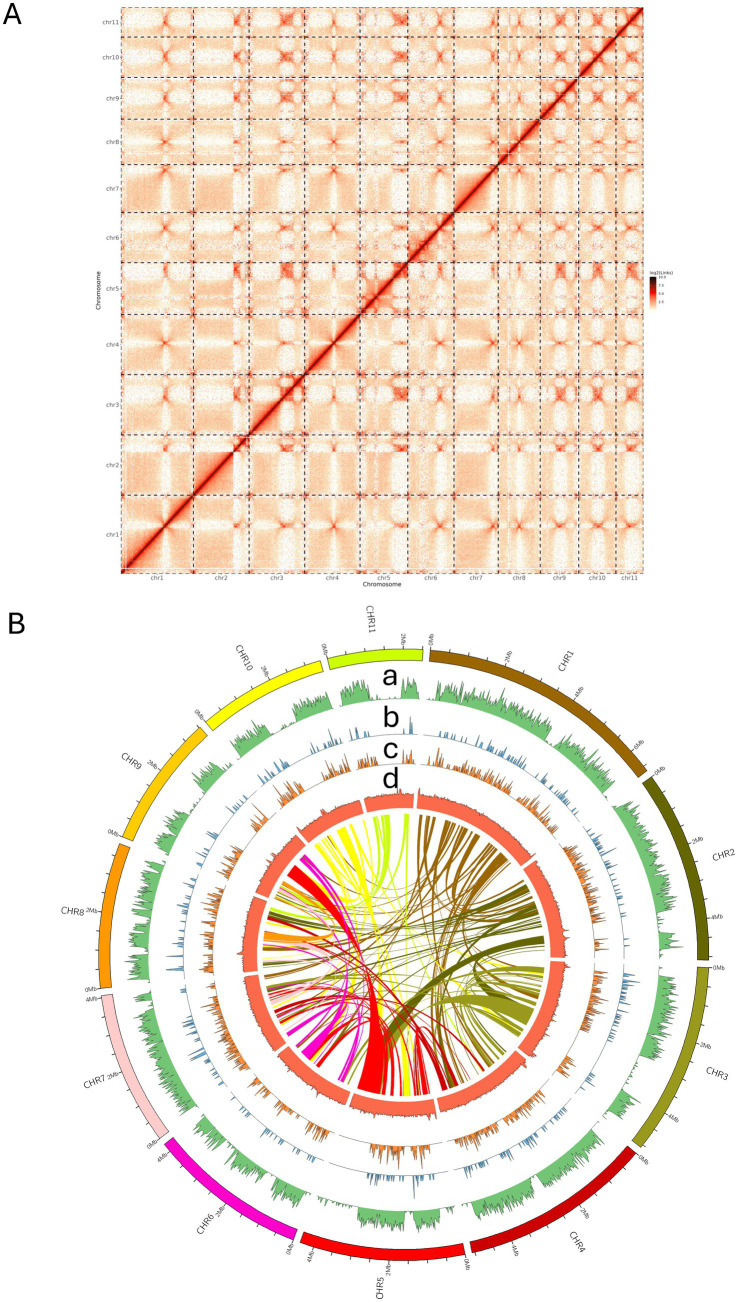
Overview of a gap-free genome assembly and annotation of *Rhizopus stolonifer* strain PRFJ02. **(A)** Hi-C contact maps showing the interaction matrices between the 11 chromosomes of *Rhizopus stolonifer* isolate PRFJ02. Colour scale indicates contact intensity between two genomic regions in a log_2_ scale. Red indicates a high contact intensity while white or yellow indicates a low contact intensity as in chromatin interaction frequency. **(B)** Ideograms of the 11 chromosomes of PRRFJ02 with chromosome length displayed using a 0.5 Mb interval; COG categories of each CDS on the forward strand (a); DFVF (b); CAZy (c); GC content (d); block illustration of syntenic relationships between chromosome pairs.

Based on an approximately 138-fold coverage, a 48.2 Mb genome assembly containing 21 contigs was produced for *Rhizopus stolonifer* isolate PRFJ02 using hifiasm (Table 1). Read pairs from Illumina Hi-C were filtered to obtain 49.7% of read pairs that were mapped as either singletons or to multiple locations (Supplementary Figure S2). Out of these, 70% of the read pairs have valid 3C interactions (*p* < 1×10^−5^), based on alignment to the Restriction enzyme-digested fragments (Supplementary Figure S3). Hi-C contact map for *R. stolonifer* isolate PRFJ02 shows that fungus carries 11 chromosomes (Figure 2A). Each axis represents the 11 assembled chromosomes, ordered and oriented based on Hi-C contact density. The strong diagonal signals represent frequent intrachromosomal contacts, which decay with genomic distance and reflect the physical folding of individual chromosomes within the nucleus. The enriched off-diagonal interaction hotspots correspond to interchromosomal contacts, which can indicate associations between specific genomic regions, such as those involved in centromere clustering. A total of 21 telomere regions containing the 5’-ACAACC-3′ repeats were identified using the telomere identifier ‘tidk’ (Brown et al., 2025) on the 11 chromosomes of PRFJ02 (Supplementary Figure S4).

**Table 1:** Assembly statistics for *Rhizopus stolonifer* isolate PRFJ02.

Assembly statistics	Primary assembly	Hi-C scaffolded assembly
Total length (Gbp)	6.6^a^, 6.8^b^, 4.1^c^	6.6^a^, 6.8^b^, 4.1^c^
Coverage (fold)	138^d^	138^d^
Assembly size (bp)	48,208,123	48,208,381
No. of contigs (or scaffolds)	21	18
Maximum contig length (bp)	6,159,681	6,651,178
N50 contig (or scaffold) length (bp)	4,233,866	4,366,636
Contig L50 (counts)	5	5
Contig L90 (counts)	10	10
GC content (%)	35.9	35.9
BUSCO coverage (%)	98.9	98.9
Total no. of BUSCOs	1,614	1,614
Complete	1,596	1,596
Duplicated	99	99
Fragmented	10	10
Missing	8	8
Gene model		
Total number of genes	NA	11,885
Number of protein-coding genes	NA	11,737
Number of rRNA genes	NA	87
Number of tRNA genes	NA	398

^a^PacBio HIFI reads, ^b^Illumina reads, ^c^Hi-C Illumina reads. ^d^This coverage refers to the PacBio sequence data relating to the de novo PacBio assembly.

The Hi-C scaffolding was further validated using the Chromap + YaHS and Chromap + SALSA2 pipelines. The initial contig assembly already contained telomere-to-telomere contigs for chromosomes 3–11 (Supplementary Figure S5). Genome-wide dot-plot self-alignment showed a clear 1:1 relationship, indicating the absence of chimeric regions. Only contigs corresponding to chromosomes 1 and 2 required scaffolding (Supplementary Figure S5). Chromosome 1 was initially represented by two telomere-containing contigs. Both the original Chromap + HiC-Pro scaffolding and Chromap + YaHS joined these contigs to form a single chromosome, whereas SALSA2 did not (Supplementary Table S2). Chromosome 2 contained one telomere and was scaffolded from one large and three smaller contigs using HiC-Pro. YaHS recommended joining only two contigs, while SALSA2 suggested further splitting the assembly into five scaffolds (Supplementary Table S2). We retained the original HiC-Pro configuration for chromosomes 1 and 2 because it produced the highest BUSCO completeness scores (Supplementary Table S3). In addition, no Hi-C signal linked these regions to other pseudochromosomes, and therefore, the contigs were not reassigned in any scaffolding run. This visualisation supports the correct chromosome-scale structure for each chromosome and confirms the near-completeness of the *R. stolonifer* PRFJ02 assembly.

Nuclear genome of PRFJ02 was assembled into primary contigs with N50 of 4.2 Mb (Table 1; Supplementary File 1). Genome completeness analysis showed a 98.9% BUSCO coverage (Table 1). The PRFJ02 genome encoded 11,885 genes across 11 chromosomes, with an average gene length of 1,530 bp (Figure 2B). GC content averaged 35.2% across the genome. A total of 36,917 tandem repeats mostly micro- and minisatellite DNAs were masked using TRF. A total of 19,033,939 bp sequence was masked using RepeatMasker which occupied 39.49% of the total genome sequence (Figure 2B; Supplementary Table S4). Retroelements, LTR elements, and DNA transposons occupied 3.34, 2.92 and 2.46% of the total repeat sequence, respectively. Eighty-seven rRNA genes and 398 tRNA genes were detected, which occupied 0.25 and 0.06% of the PRFJ02 genome (Supplementary Table S5).

### 3.3 Functional annotations of PRFJ02

A total of 11,737 protein-coding genes were annotated in PRFJ02 (Table 1, Additional File 1). GO term classification for PRFJ02 revealed that the most enriched Biological Process terms were cellular nitrogen compound metabolic process (1934), biosynthetic process (1849), small molecule metabolic process (1201), transport (1121), and catabolic process (959) (Supplementary Figure S6). For the Cellular Component category, the top five terms included cell (3556), intercellular (3642), organelle (2681), cytoplasm (2314), and protein complex (811). In the Molecular Function category, the most abundant terms were ion binding (2415), DNA binding (647), oxidoreductase activity (597), kinase activity (566), and RNA binding (465).

KEGG annotation of the *R. stolonifer* PRFJ02 genome showed protein-coding genes commonly associated with metabolism, particularly carbohydrate, amino acid, lipid, and energy metabolism, as well as secondary metabolite biosynthesis (Supplementary Figure S7). Genes related to Genetic Information Processing were also abundant, spanning translation, replication/repair, and protein and folding. Signal transduction formed the largest component of Environmental Information Processing, while additional genes were linked to Cellular Processes, including transport and catabolism. Overall, the KEGG classification showed that PRFJ02 possesses a strong metabolic capacity and regulatory flexibility. The three most abundant KOG categories are Translation, ribosomal structure and biogenesis, Post-translational modification, protein turnover, chaperones, and Signal transduction mechanisms (Supplementary Table S6).

### 3.4 Comparative analysis using conserved orthologues

**Figure 3:**
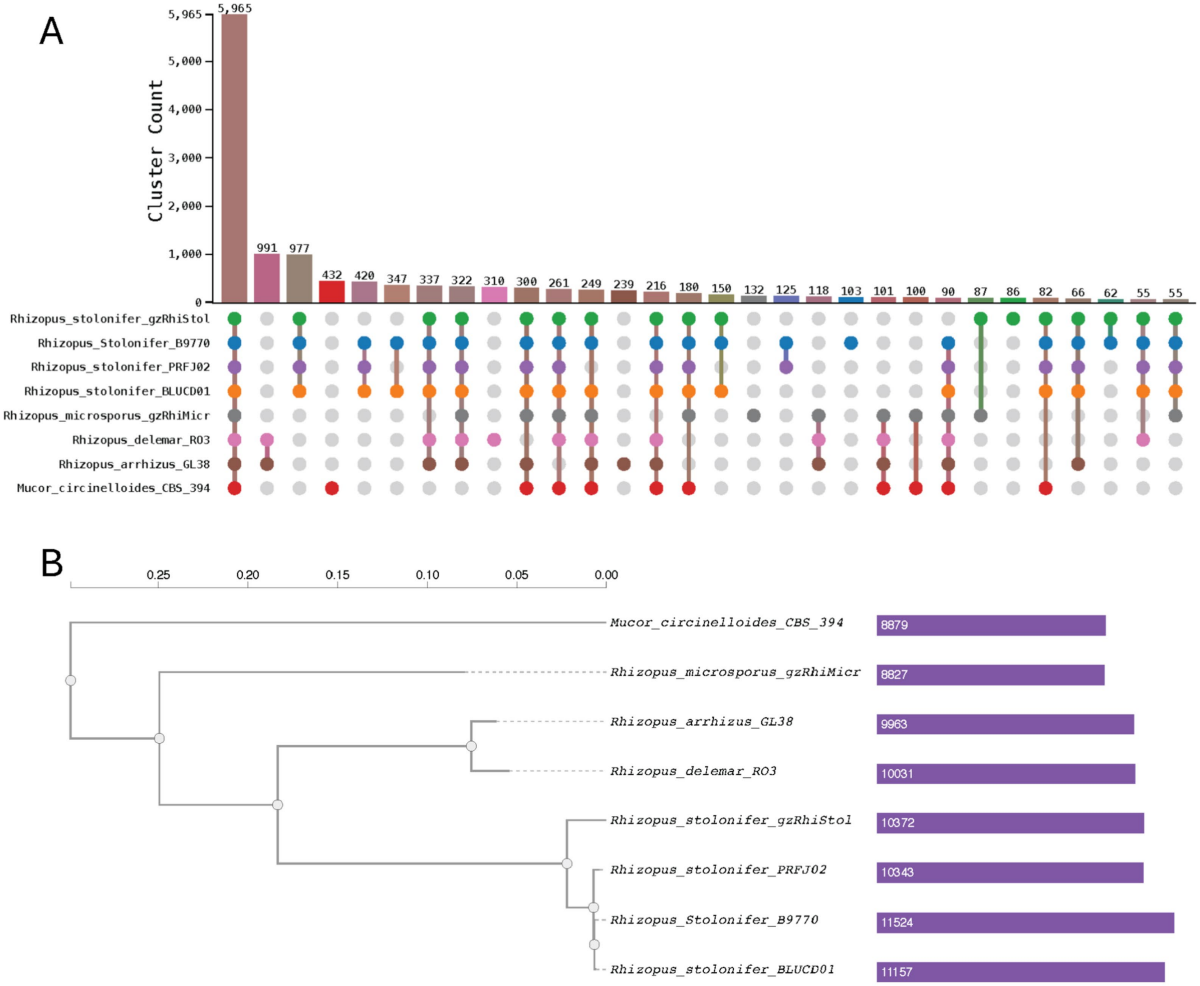
Orthologous gene cluster analysis. **(A)** Upset plot for orthologous gene clusters showing each genome and genome pairs. **(B)** Phylogenetic tree generated using FastTree2. The numbers indicate the number of orthogroups detected in each genome. Branch lengths are scaled to represent the estimated number of amino-acid substitutions per site.

Protein clustering analysis using OrthoVenn3 revealed 14,913 conserved orthologous clusters in *R. stolonifer* isolates PRFJ02, gzRhiStol, B9770, BLUCD01, and *R. microsporus* isolate gzRhiMicr, *R. delemar* isolate RO3, *R. arrhizus* isolate GL38, and *Mucor circinelloides* isolate CBS 394.68 (Supplementary Table S7). A core orthologous gene set of 5,965 was evident (Figure 3A), of which 3,055 appeared to be single-copy orthologues amongst the eight genomes (Supplementary Table S7). Whole-genome phylogeny based on single gene clusters showed that all 4 *R. stolonifer* isolates clustered together as a single phylogroup which is evolutionally distinct from the other lineage that gave rise to *R. arrhizus* and *R. delemar* (Figure 3B). The ancestorial split giving rise to this node also gave rise to *R. microsporus*, which is relatively more divergent to *R. stolonifer*, *R. arrhizus* and *R. delemar*. The topology of this tree agrees with the one published in another study (Gryganskyi et al., 2018). The four *R. stolonifer* strains exclusively shared 977 gene clusters (Figure 3A). The most abundant GO terms are enriched for sporulation (GO:0030435, *p* = 0.0), signal transduction (GO:0007165, *p* = 0.0), regulation of transcription from RNA polymerase II (GO:0006357, *p* = 0.0), translation (GO:0007165, *p* = 0.0), and signalling (GO:0023052, *p* = 0.0) (Supplementary Figure S8).

To explore host association, we performed gene orthology analysis on the four *Rhizopus stolonifer* strains. A total of 9,073 clusters were detected across all four genomes (Supplementary Figure S9A) and 7,903 of which were single clusters (Supplementary Table S8). CAFE5 phylogenetic tree shows that the tomato infecting strain BLUCD01 is more closely related to B9770, which was isolated from a contaminated product, than to our PRFJ02 isolate from passion fruit (Supplementary Figure S9B). The isolate gzRhiStol1 is phylogenetically distant to the rest. PRFJ02 has 39 unique gene clusters and is enriched for ATP synthesis coupled electron transport (4 genes, *p* = 1.26E-07), electron transport proton transport (2 genes, *p* = 0.0001) at *p* < 0.001 level. BLUCD01 has 16 unique gene clusters but they are not enriched with any GO terms. Both plant pathogens (PRFJ02 and BLUCD01) share 65 gene clusters, exclusive of the other 2 isolates. These gene clusters are enriched for GO terms in glutathione transferase activity (4 genes, *p* = 9.74E-05) and galactose catabolic process (2 genes *p* = 0.0005).

In line with these lineage-specific patterns, gene family evolution across the isolates revealed substantial differences in lineage-specific expansion and contraction among the *Rhizopus stolonifer* isolates. PRFJ02 exhibited the greatest gene family contraction, losing 910 orthogroups, followed by BLUCD01 with 248 losses (Supplementary Figure S9B). In contrast, both genomes showed relatively limited gene family expansion, with only 22 and 65 orthogroups gained in PRFJ02 and BLUCD01, respectively. B9770 displayed a more balanced pattern of gene family turnover, with 150 gains and 170 losses, whereas gzRhiStol1 showed net expansion, gaining 74 orthogroups while losing only 25. Overall, these patterns suggest that host-associated lineages may have evolved primarily through gene loss, consistent with adaptive specialisation to particular plant hosts.

### 3.5 Analysis of effector repertoire

**Figure 4:**
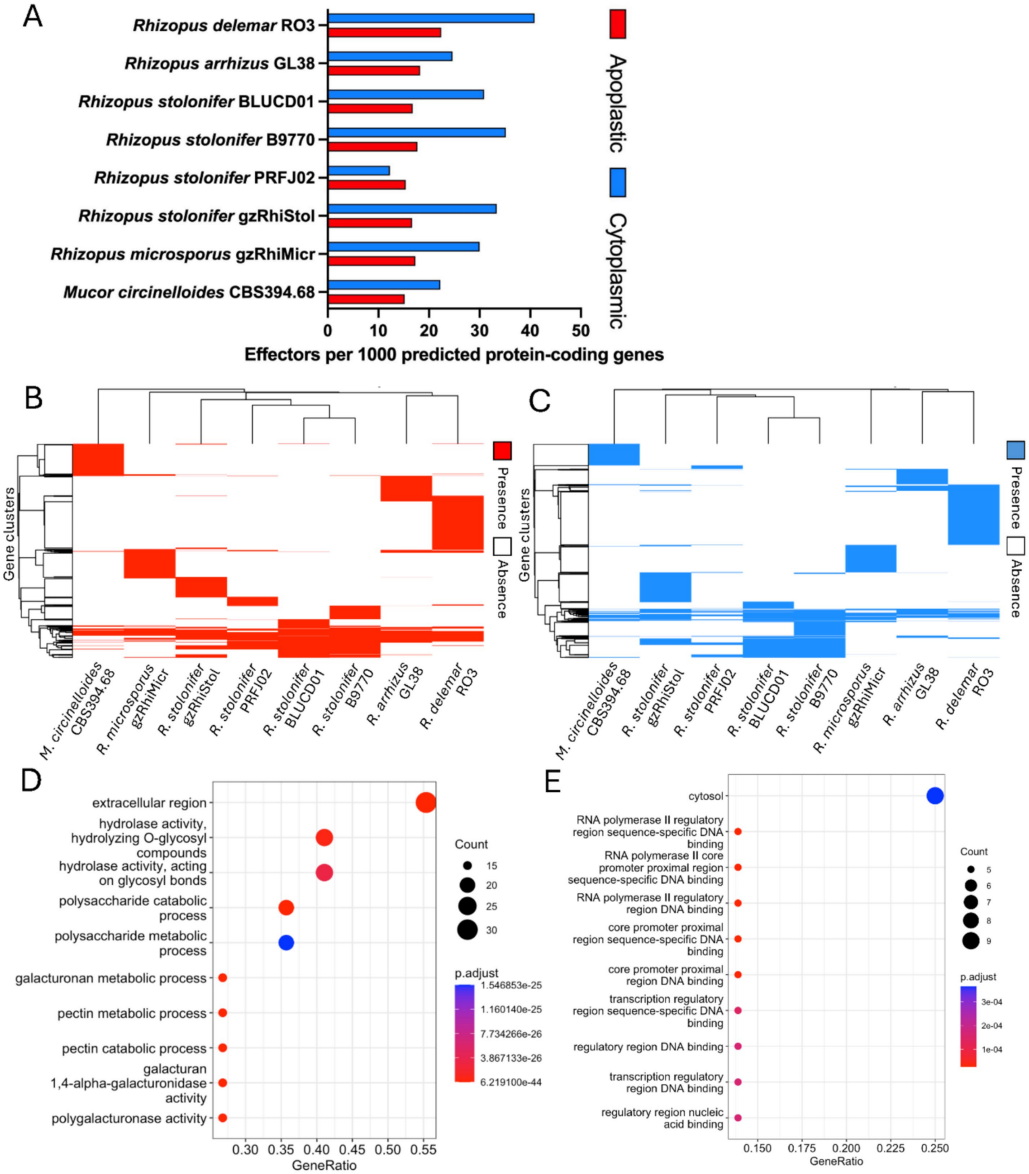
Genome-wide analysis of candidate effector gene repertoires. **(A)** Predicted number of apoplastic and cytoplasmic candidate effector genes in the *Rhizopus* genomes. Heatmap of genome presence/absence across MMseq2 **(B)** apoplastic and **(C)** cytoplasmic effector gene clusters. Hierarchical clustering of genomes (*x*-axis) and gene clusters (*y*-axis) is shown by dendrograms. Gene ontology enrichment analysis of **(D)** apoplastic and **(E)** cytoplasmic PRFJ02 candidate effectors, found to be commonly shared amongst all four *Rhizopus stolonifer* genomes.

Effector profiling showed that *R. stolonifer* PRFJ02 possessed 183 and 146 apoplastic and cytoplasmic effectors, respectively (Supplementary Figure S10). Cytoplasmic effector numbers are higher than that of apoplastic effectors for all strains but PRFJ02. After normalising for the total number of protein-coding genes per genome, PRFJ02 exhibited the lowest number of both effector types among the analysed genomes, with its cytoplasmic effector repertoire being ≤50% of that observed in the other genomes (Figure 4A).

The heatmap of effector gene presence/absence across MMseq2 clusters reveals the distribution of shared and unique apoplastic or cytoplasmic effector gene families among the eight fungal genomes (Figures 4B,C). The relative positions of the genomes in this hierarchical clustering are consistent with the phylogenetic tree inferred from orthologue clusters. Dominant presence of unique clusters or blocks of effector genes in each genome suggests a divergence of virulence repertoire amongst these genomes. This is particularly evident as you move away from the central clusters to other Rhizopus species and *Mucor circinelloides*. The exclusive set common to the 4 *R. stolonifer* genomes appeared relatively small, containing only 14 (apoplastic) and 22 (cytoplasmic) gene clusters (Supplementary Figure S11) and therefore, no significant GO term enrichment was detected using the PRFJ02 genes defined by these clusters and its GO terms. Instead, functional enrichment analysis was performed on the broader set of effector clusters shared among the *R. stolonifer* genomes to identify conserved functional signatures within the effector repertoire. The shared set, indicative of functional effector repertoire, identified significantly enriched GO terms in the PRFJ02 genome (*p* < 0.05). The most enriched GO categories in the apoplastic effector set included terms associated with the extracellular region, hydrolase activity, and polysaccharide-related processes such as galacturonan and pectin metabolism (Figure 4D). In contrast, the cytoplasmic effector set was enriched for cytosolic localisation and GO terms related to the regulation of RNA polymerase II transcription and sequence-specific DNA binding involved in transcriptional regulation (Figure 4E). The two plant-infecting *R. stolonifer* strains, PRFJ02 and BLUCD01, shared few effector clusters that were exclusive to these genomes when compared with the remaining six genomes (Supplementary Figure S11). Overall, we found no evidence to suggest that host association in passion fruit and tomato infecting strains is linked to unique effector gene repertoires. Instead, an effector gene set common to the *R. stolonifer* strains was observed, suggesting that adaptation to specific environments may occur through other mechanisms, such as gene loss or variation in CAZyme composition.

### 3.6 CAZyme profiling and secretome prioritisation

**Figure 5:**
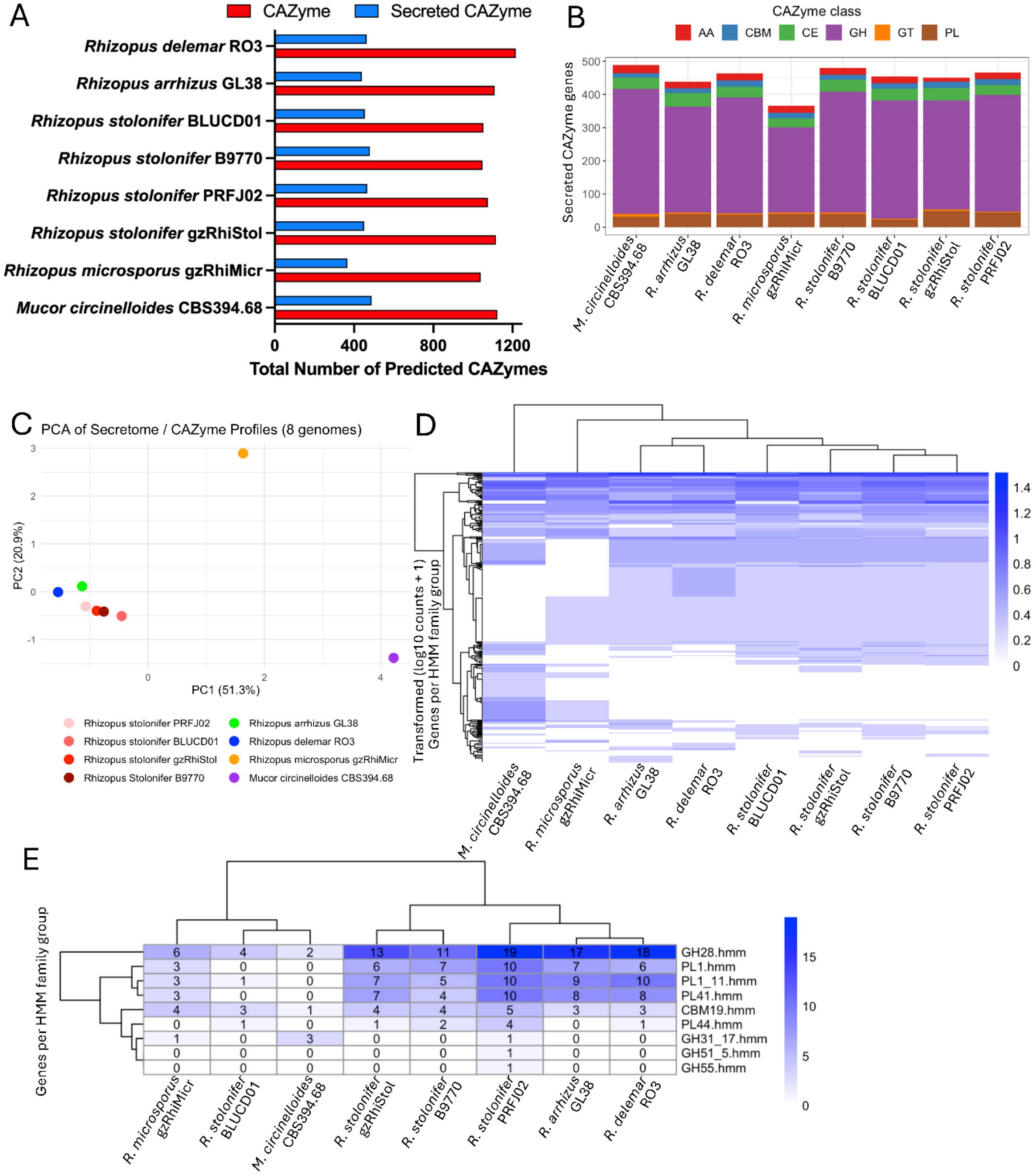
Analysis of CAZymes prioritised for secretion in the eight *Rhizopus* genomes. **(A)** Total number of CAZymes with and without a predicted signal peptide in each genome. **(B)** Six CAZyme classes and their numbers detected in the secreted CAZymes of each genome. AA = Auxiliary activities; CBM = Carbohydrate binding modules; CE = Carbohydrate esterases; GH = Glycoside hydrolases; GT = Glycosyltransferases; PL = Polysaccharide lyases. **(C)** Principal component analysis using log-transformed (log_10_(count + 1)) counts of HMM families across all genomes. **(D)** Whole-genome hierarchical clustering of log-transformed CAZymes prioritised for secretion across all eight genomes. **(E)** HMM families that are predominant in *Rhizopus stolonifer* PRFJ02 relative to either the full set of eight genomes or the *four R. stolonifer* genomes.

The total number of predicted CAZymes varied across the eight fungal genomes, with *Rhizopus* species generally containing between ~800 and 1,100 CAZyme-encoding genes (Figure 5A). The subset of CAZymes predicted to be secreted was considerably smaller than the total CAZyme complement in each genome. The predicted number of secreted CAZymes appear varied among strains. PRFJ02 displayed a secreted CAZyme set comparable in size to the other *R. stolonifer* strains.

Analysis of CAZyme classes prioritised for secretion revealed that glycoside hydrolases (GH) dominated the secreted CAZyme profiles across all genomes, followed by auxiliary activity (AA), carbohydrate-binding modules (CBM), and polysaccharide lyases (PL), whereas carbohydrate esterases (CE) and glycosyltransferases (GT) were represented by fewer genes (Figure 5B). The overall pattern of class abundance was largely consistent across species. Minor differences in the composition of CAZyme classes were observed among genomes of the *R. stolonifer* strains.

Principal component analysis (PCA) of secreted CAZyme repertoires revealed clear genome-level separation (Figure 5C). PC1 captures the major differences, likely driven by highly abundant HMM families that vary significantly among genomes. PC2 captures secondary variation, highlighting differences in less abundant but distinctive HMM families. The *M. circinelloides* and *R. microsporus* genomes were distinct from all other Rhizopus genomes, indicating divergence in enzyme family repertoires. The four *R. stolonifer* genomes clustered closely together, with *R. arrhizus* and *R. delemar* positioned nearby within the broader *Rhizopus* cluster.

The genome-wide CAZyme and secretome analysis across eight fungal genomes was visualised as a heatmap representing the number of secreted proteins associated with each HMM family (Figure 5D). The distribution and abundance of CAZyme families showed that many HMM families are represented by a low number of genes across the eight genomes. Copy number variation is evident, with most differences observed between the four *R. stolonifer* genomes and the other species, while the four *R. stolonifer* genomes themselves exhibit relatively similar CAZyme repertoires. To examine genome-specific variation, HMM families were identified in which *R. stolonifer* PRFJ02 had the highest number of genes relative to the other seven genomes, as well as families in which PRFJ02 was dominant compared only to the other three *R. stolonifer* genomes. This analysis identified nine HMM families with overrepresented gene counts in PRFJ02 (Figure 5E). These included genes from the glycoside hydrolase family GH28, polysaccharide lyase families PL1, PL1_11, and PL41, and the carbohydrate-binding module family CBM19. In addition, PRFJ02 contained one gene each from glycoside hydrolase families GH31_17, GH51_5, and GH55, which were absent in the other *R. stolonifer* genomes (Figure 5E).

## 4 Discussion

*Rhizopus stolonifer* is a rapid-growing necrotrophic pathogen capable of colonising a wide range of plant tissues, particularly those with high sugar and moisture content. In this study, we isolated *R. stolonifer* PRFJ02 from infected passion fruit flowers and generated a chromosome-scale genome assembly to characterise its genomic features. The observed morphology of PRFJ02, including rhizoids, stolons, and sporangia producing abundant sporangiospores, is consistent with previous descriptions of *R. stolonifer* (Kwon and Lee, 2006; Liu et al., 2024), and the field symptoms reflect the rapid tissue colonisation typical of soft rot diseases (Petrasch et al., 2019).

The high-quality genome assembly of PRFJ02 provides a robust resource for genomic analyses. The assembled genome size (~48 Mb) is consistent with previously reported genomes of *Rhizopus* species and other mucoralean fungi (Gryganskyi et al., 2018; Mesquida-Pesci et al., 2025). Functional annotation further revealed a broad set of genes involved in metabolism, signal transduction, and protein processing. This indicates that *R. stolonifer* possesses extensive metabolic capacity that may support rapid growth and colonisation of host tissues.

Comparative genomic analysis across related *Rhizopus* species revealed a conserved core gene set alongside lineage-specific gene clusters. PRFJ02 showed notable gene family contraction relative to other genomes, suggesting variation in gene content among isolates. Gene family contraction is a common feature of evolutionary dynamics in fungal plant pathogens and may accompany adaptation to specific ecological niches or host environments (Raffaele and Kamoun, 2012; Gladieux et al., 2014). In PRFJ02, this is reflected by a reduced representation of conserved orthogroups relative to the other *R. stolonifer* genomes analysed.

Effector prediction and comparative analysis showed that most effectors are genome-specific, with only a limited number shared among the four *R. stolonifer* isolates. The relatively small and weakly conserved core effector sets suggest that effector repertoires are highly variable across genomes. Only a small subset of effectors was shared exclusively among plant-infecting strains, indicating that differences in effector content alone do not explain variation among these isolates. Interestingly, PRFJ02 possessed the lowest number of predicted cytoplasmic effectors among the analysed strains, despite being isolated from a plant host. Cytoplasmic effectors are commonly associated with the suppression of host immune responses in biotrophic and hemibiotrophic pathogens (Sperschneider et al., 2022), whereas necrotrophic fungi deploy effectors that contribute to host cell death and necrotrophic infection strategies (Shao et al., 2021; Wang et al., 2014). The reduced number of such effectors in PRFJ02 suggests that *R. stolonifer* may rely less on sophisticated host immune suppression mechanisms and more on direct tissue degradation during infection. This observation is consistent with the necrotrophic lifestyle of *R. stolonifer*, where hot cell death is advantageous (van Kan, 2006; Shao et al., 2021).

The analysis of secreted carbohydrate-active enzymes revealed that glycoside hydrolases were the most abundant class across all analysed genomes. These enzyme classes are involved in the breakdown of major plant cell wall components including cellulose, hemicellulose, and pectin (Lombard et al., 2014; Zhao et al., 2013). PCA analysis of secreted CAZyme repertoires showed that the four *R. stolonifer* genomes share broadly similar enzymatic profiles, while *M. circinelloides* and *R. microsporus* displayed more divergent repertoires. In PRFJ02, several CAZyme families, including glycoside hydrolase family GH28 and polysaccharide lyase families PL1, PL1_11, and PL41, were enriched. The glycoside hydrolase superfamily includes enzymes such as polygalacturonases that hydrolyse the homogalacturonan backbone of pectin (Lombard et al., 2014; Jayani et al., 2005), while polysaccharide lyases cleave pectic polysaccharides via *β*-elimination, primarily targeting de-esterified pectin (Collmer and Keen, 1986; Jayani et al., 2005). Pectin constitutes a major structural component of plant cell walls, particularly in fruit and floral tissues, and its degradation is a key step in the maceration of host tissues during necrotrophic infection (Vorwerk et al., 2004; van Kan et al., 2006; Kubicek et al., 2014; Lorrai and Ferrari, 2021). The enrichment of secreted pectin-degrading CAZymes in PRFJ02 therefore indicates an important role for pectin degradation in the infection strategy of *R. stolonifer* in passion fruit.

## 5 Conclusion

Overall, the genomic and functional analyses presented in this study provide a comprehensive view of *R. stolonifer* PRFJ02, a causal agent of flower rot in passion fruit. The high-quality assembly and annotation enabled comparative analyses that confirmed the distinct evolutionary placement of *R. stolonifer* strain PRFJ02 within the genus and identified both conserved orthologs and isolate-specific gene clusters. Overall, the genomic analyses indicate that *R. stolonifer* relies on rapid growth and a versatile set of secreted enzymes for host colonisation, rather than a highly specialised repertoire of effectors. This resource offers a foundation for further investigation into the biology of this pathogen and lays the groundwork for future development of resistant cultivars and improved management strategies for soft-rot diseases in fruit crops.

## Data Availability

The genome assembly, and its annotation files, as well as additional data files 1–5 are available at http://www.doi.org/10.5281/zenodo.18521605. Raw Pac-bio and Illumina data are publicly available in NCBI within the respective sequence read archive ‘SRR35738426’ and ‘SRR35738425’, under Bioproject ‘PRJNA1337812’. The assembly (PRJ02.scaffolds) is deposited in NCBI genome under accession ‘JBSRNQ000000000’.
