# Supplementary material for: A chromosome-level genome assembly of Rhizopus stolonifer associated with passion fruit flower rot
Source: Front Microbiol. 2026 Apr 13;17:1757919. doi: 10.3389/fmicb.2026.1757919 (PMC13111411; doi:10.3389/fmicb.2026.1757919)
Supplement: Supplementary file 1 [file Supplementary_file_1.docx]

A chromosome-level genome assembly of *Rhizopus stolonifer* associated with passion fruit flower rot

**Jiaman Sun^1,†^**, **Ge Chen^2,†^**, **Donald M. Gardiner^3^**, **Sabrina Morrison**^3^, **Xiaonan Zhang^1^**, **Elizabeth A. B. Aitken^4^**, **Liu Yang^2,^**^*,†^ **and** **Andrew Chen^4,^**^*^**^,^**^†^


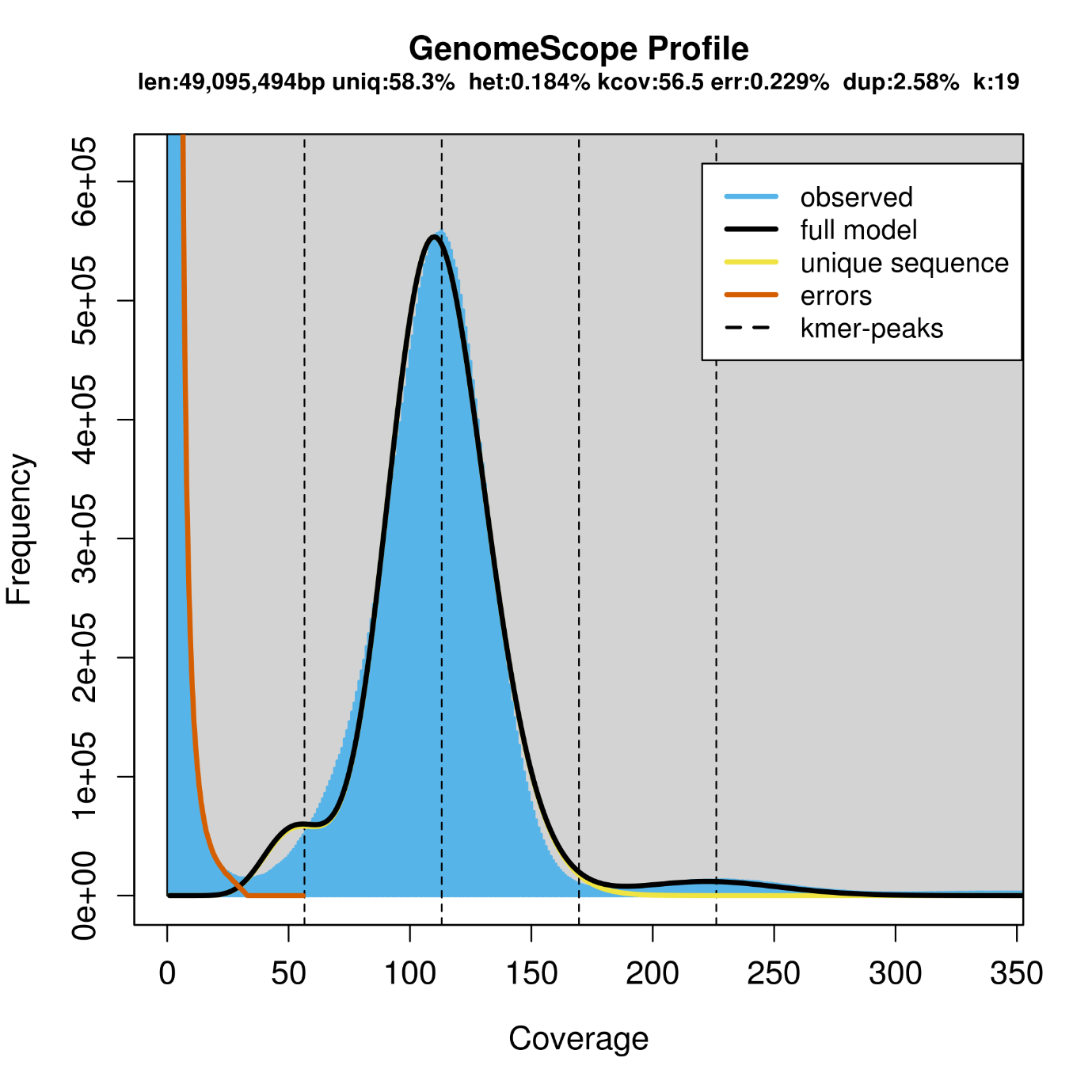


**Supplementary Figure S1.** Kmer analysis using k = 19. Genome Scope profile based on Pac-bio short-insert libraries (insert size < 1,500 bp).


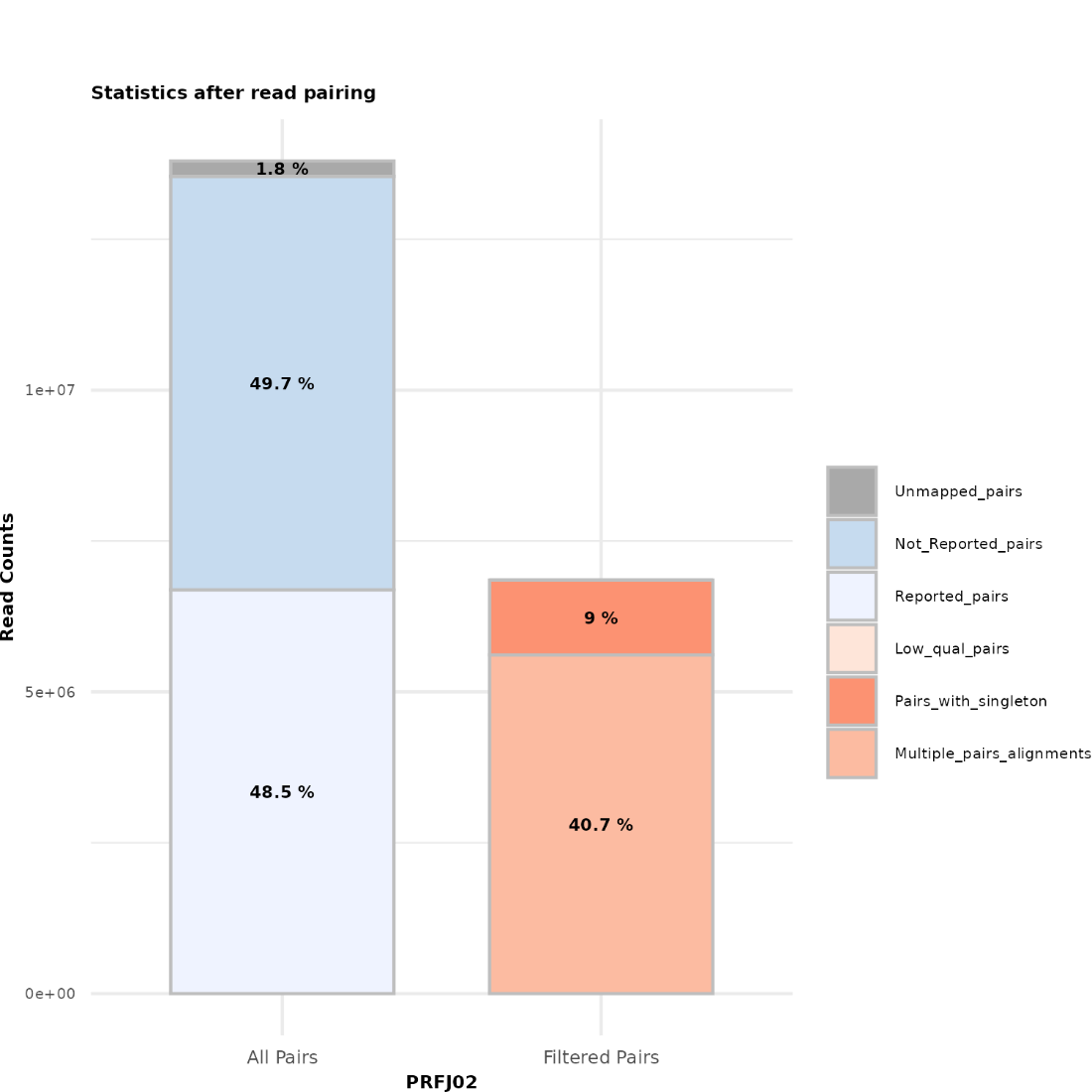


**Supplementary Figure S2.** Hi-C Illumina read pair data filtering.


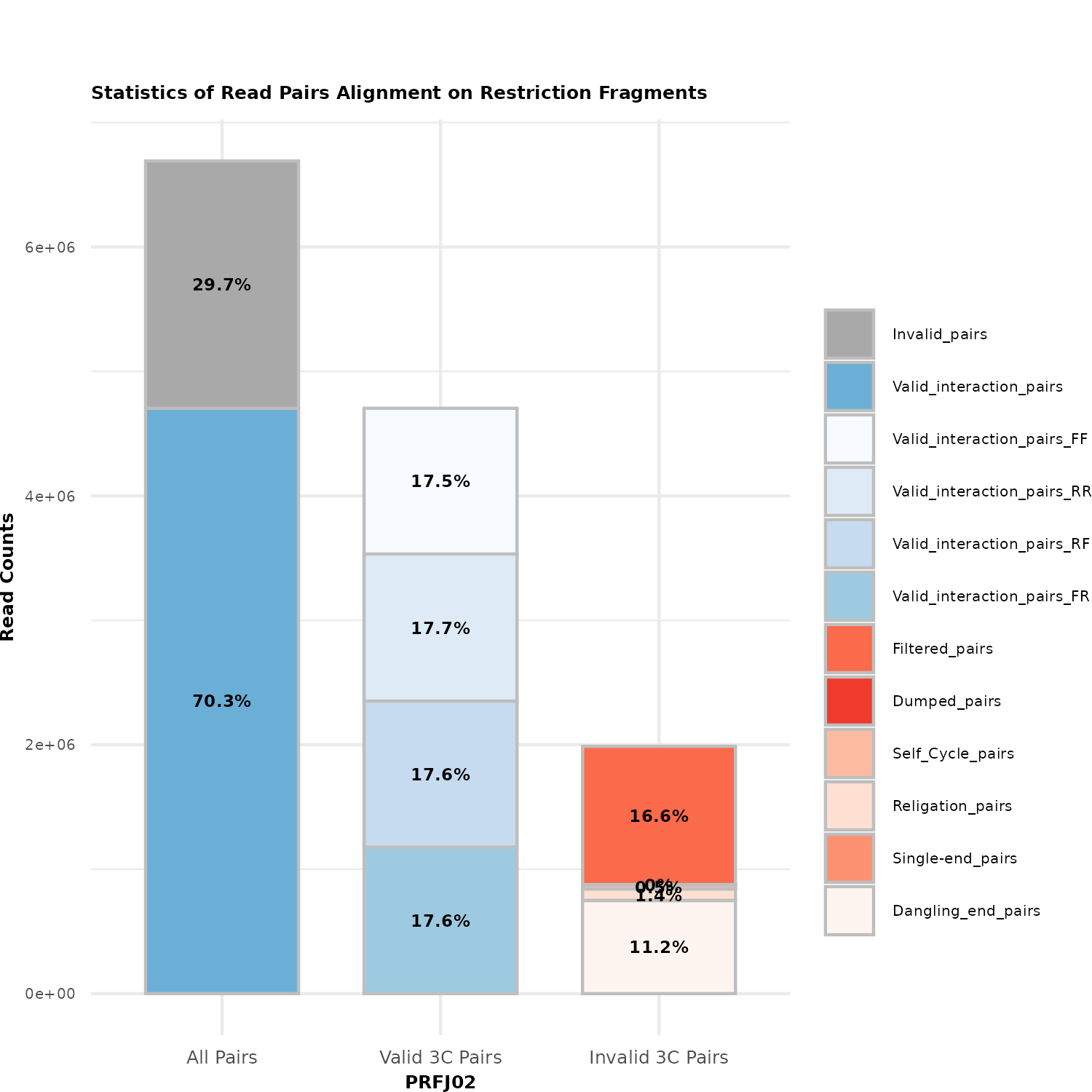


**Supplementary Figure S3.** Read pairs alignment on Restriction Fragments. Filtered read pairs were analysed to validate 3C read pairs that have interactions.





**Supplementary Figure S4**. Relative presence of telomeric repeats ‘ACAACC’ detected using the telomere identifier ‘tidk’ across all 11 chromosomes.


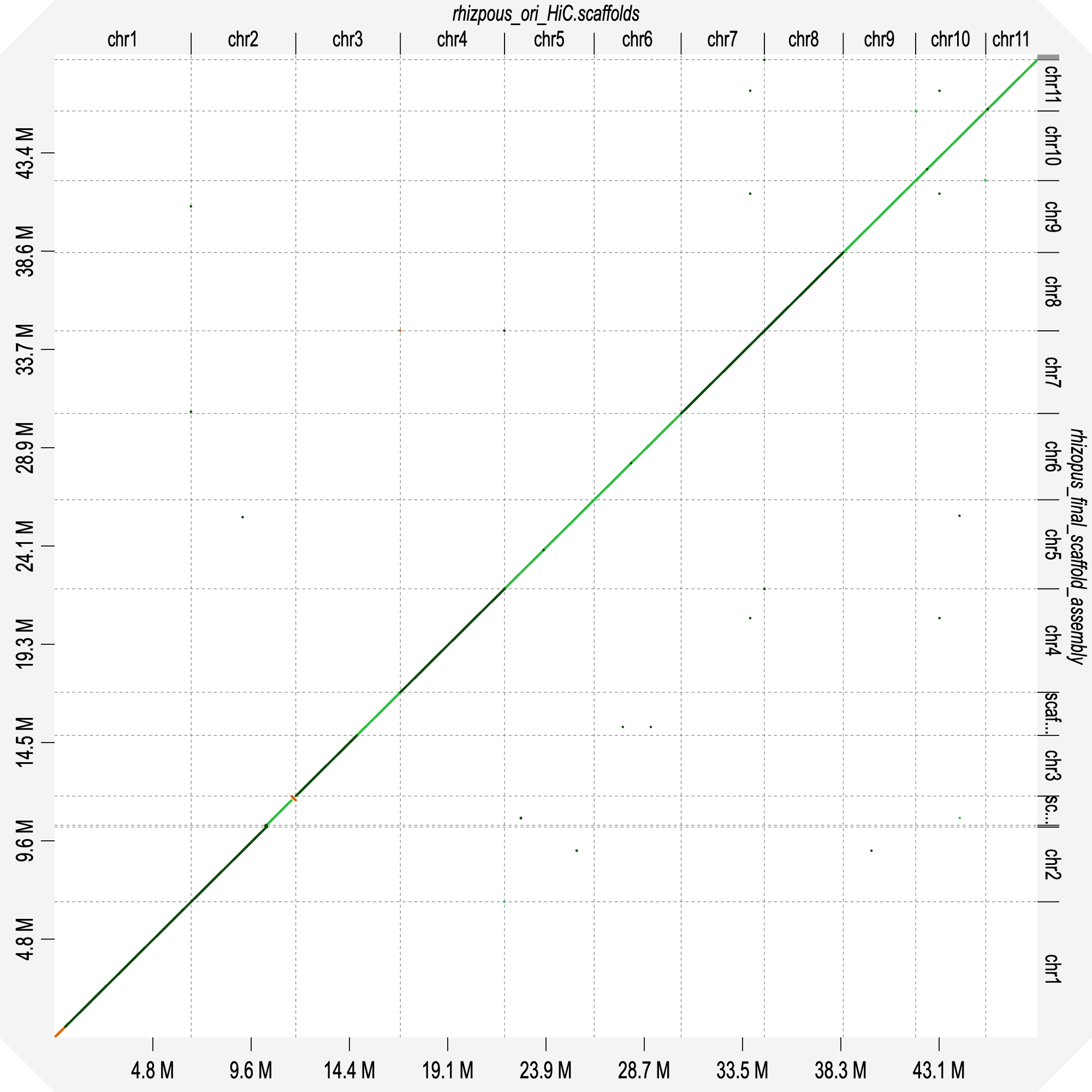


**Supplementary Figure S5**. Dotplot comparing *Rhizopus stolonifer* original Hi-C assembly (x-axis) with final scaffolded assembly (y-axis). Dotplot shows splitting on chr2 and chr3 in original assembly.


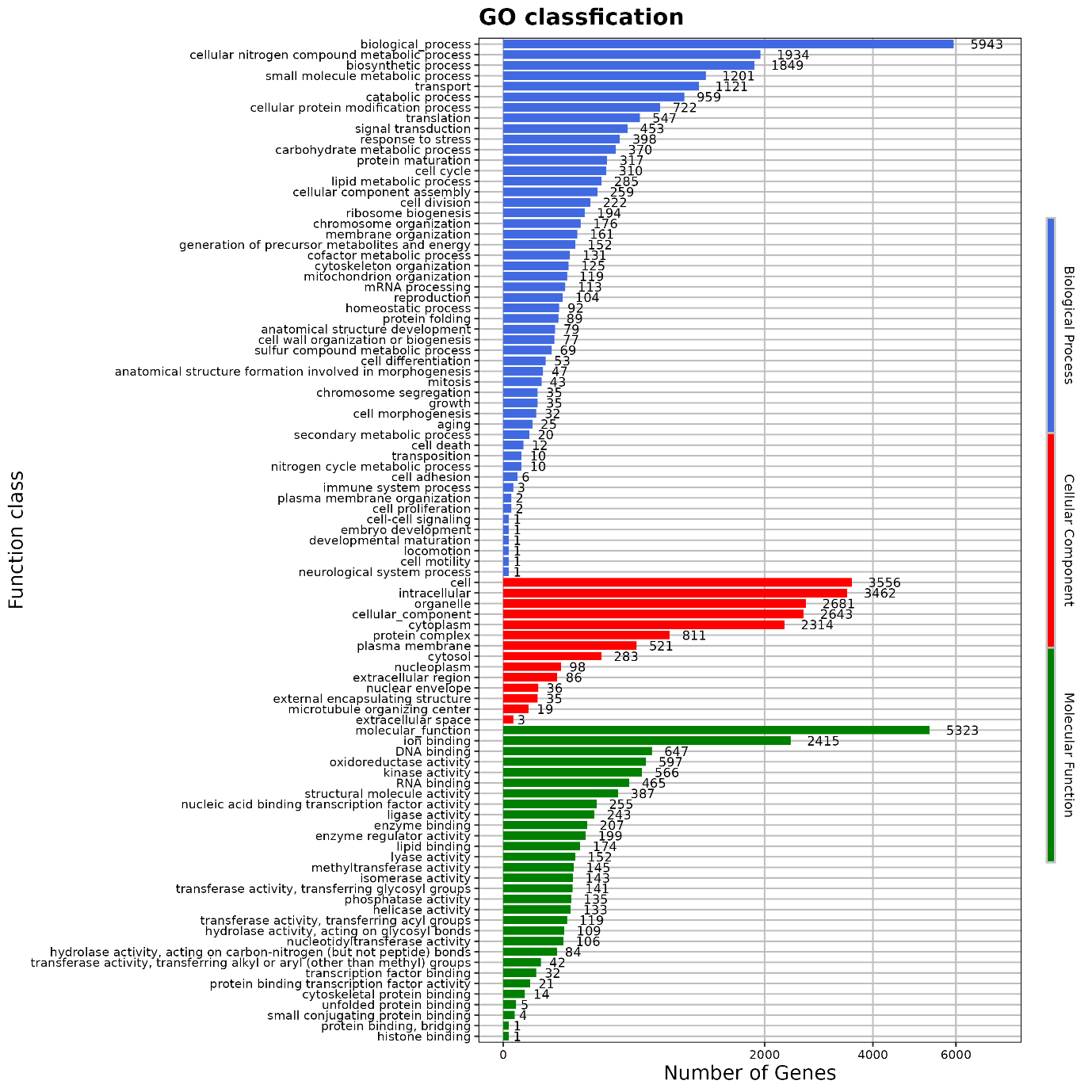


**Supplementary Figure S6.** GO term classification for the genome of *Rhizopus stolonifer* isolate PRFJ02.


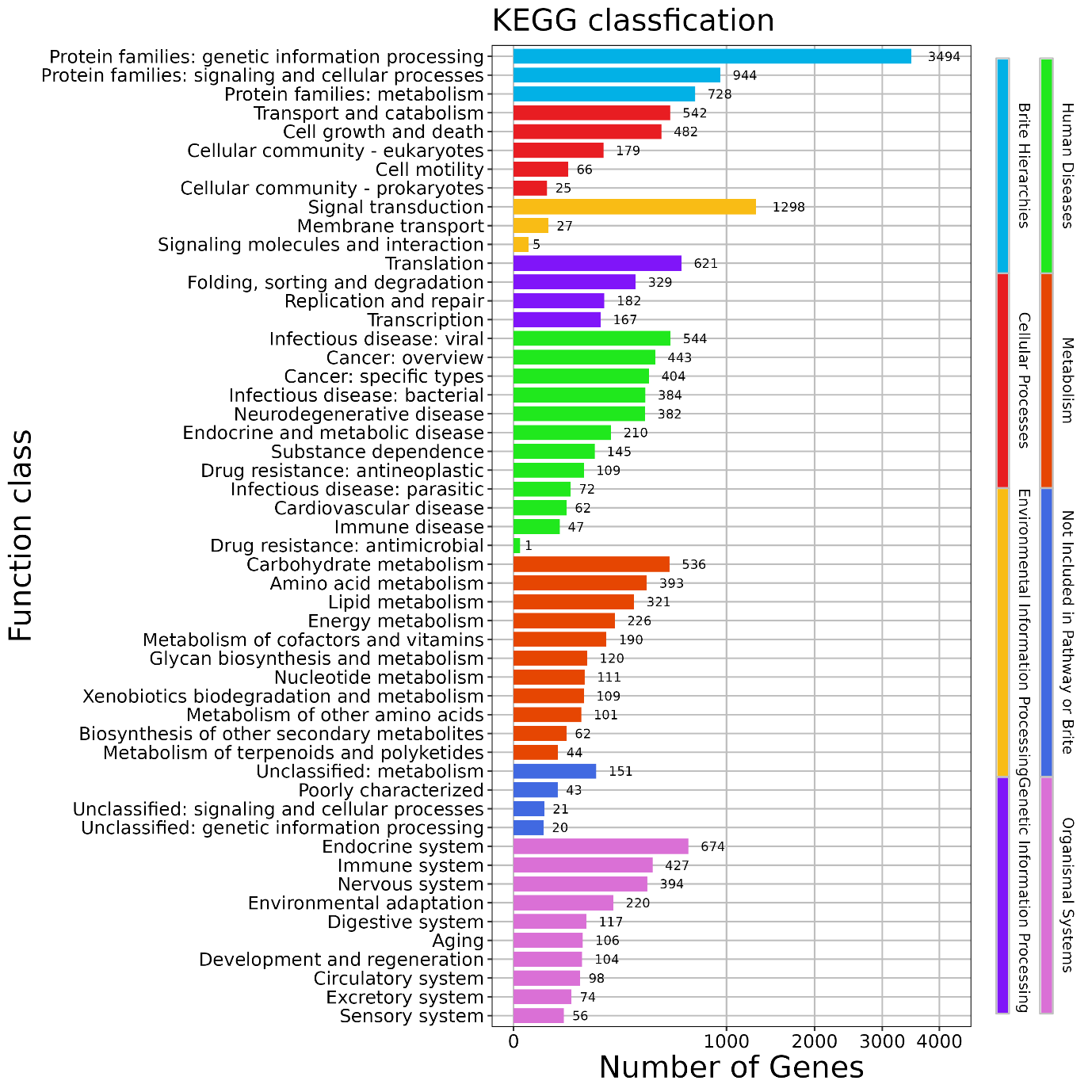


**Supplementary Figure S7.** KEGG classification for the genome of *Rhizopus stolonifer* isolate PRFJ02. Protein-coding genes of the PRFJ02 genome were classified into eight functional categories based on KEGG annotation, with each category comprising genes assigned to KO identifiers and pathway memberships.


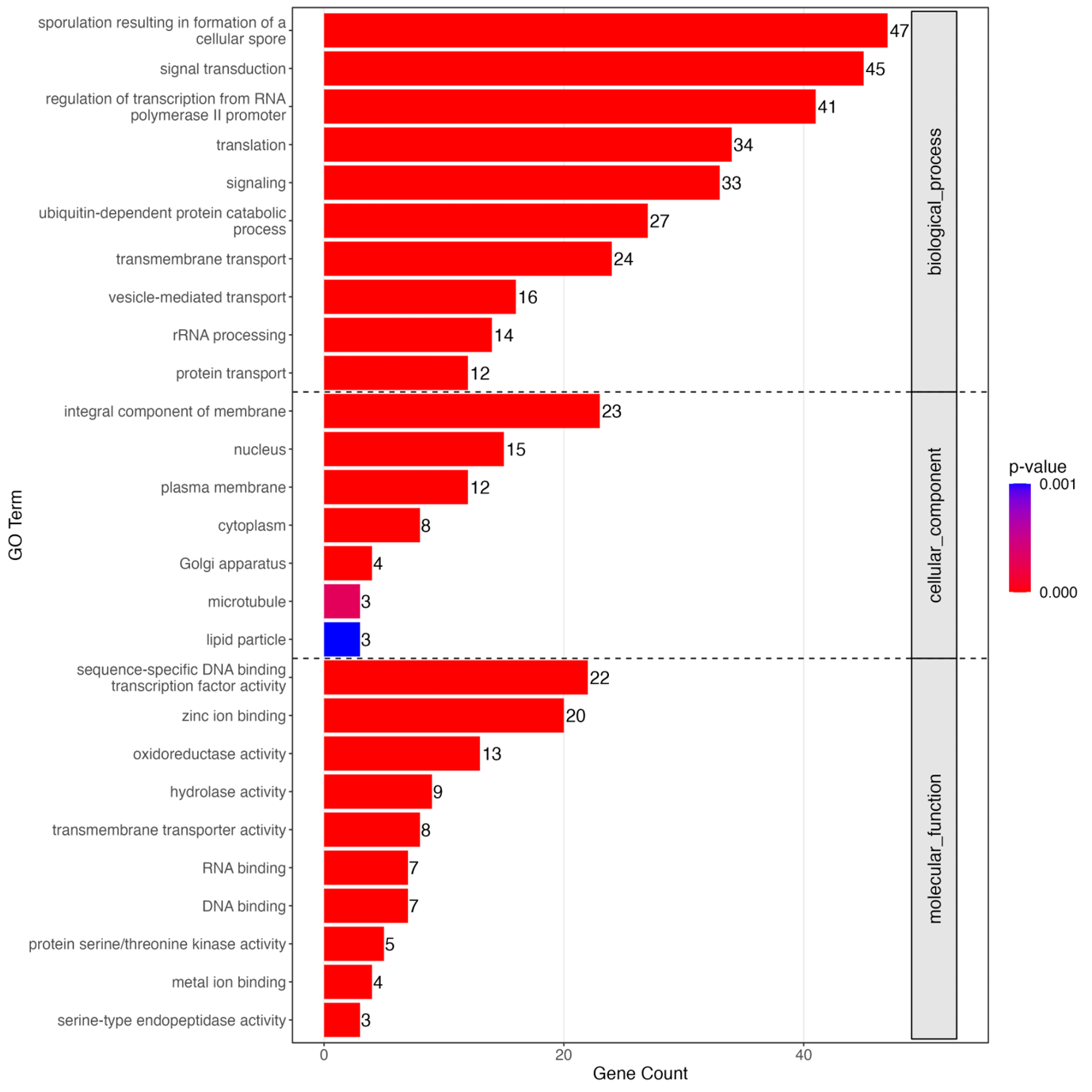


**Supplementary Figure S8.** Top 10 abundant GO terms from each GO category detected in the 977 orthologue clusters exclusive to the four *Rhizopus stolonifer* strains.


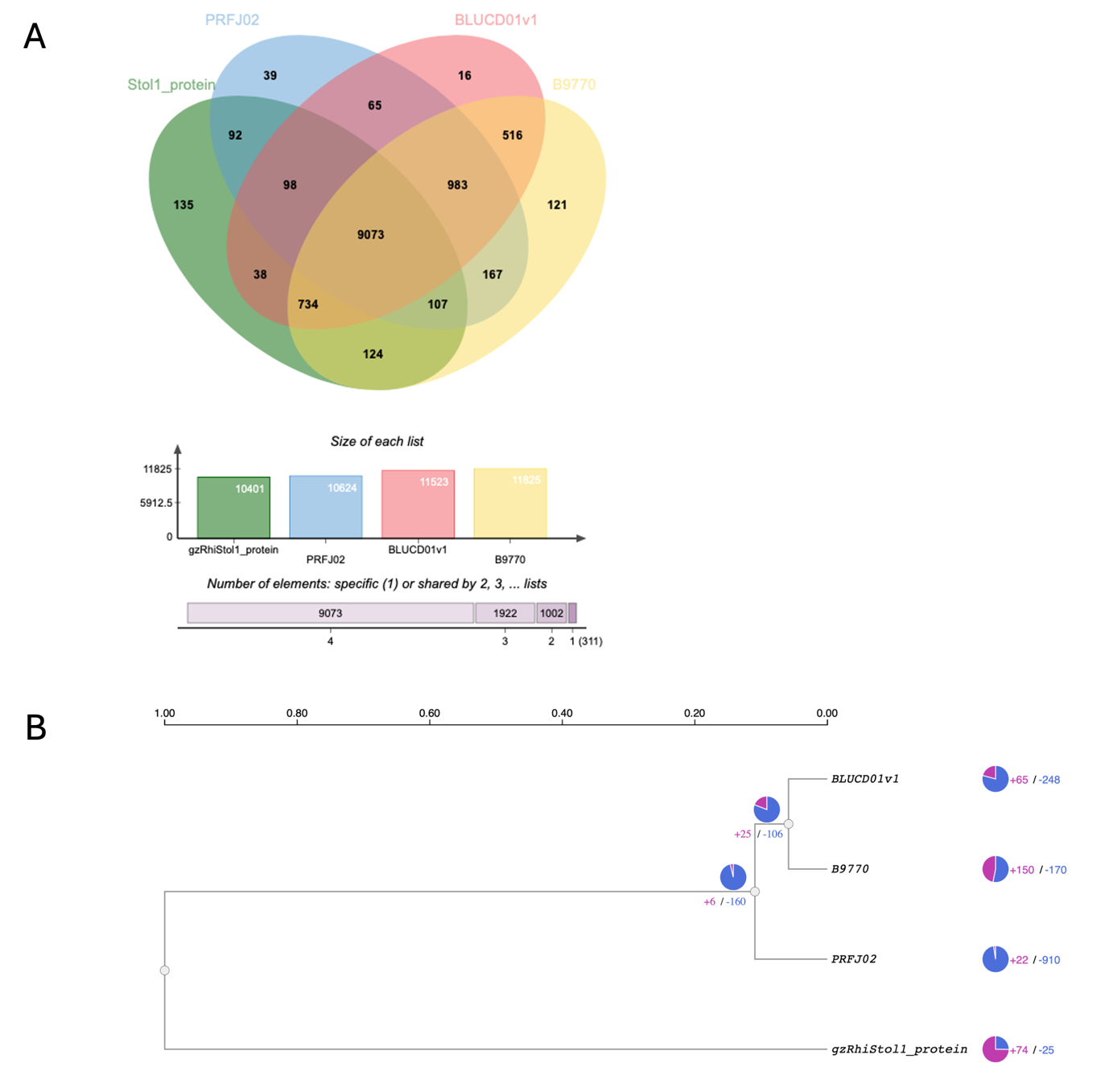


**Supplementary Figure S9**. Gene ortholog analysis amongst the four *Rhizopus stolonifer* strains. (**A**) Venn diagram depicting the shared and unique orthologous clusters across the four isolates. Numbers within overlapping regions indicate clusters shared among the corresponding isolates, whereas numbers in non-overlapping regions represent unique clusters. The colour-coded bar chart accompanying the panels corresponds to the number of orthologous gene clusters detected in each isolate. Gene clusters are distributed according to the number of lists (isolates) they are present in. (**B**) Phylogenetic tree generated with CAFE5 showing the gene family gain and losses over evolutionary time. Branch length is not estimated due to the unavailability of divergence time data at the genus/species level.


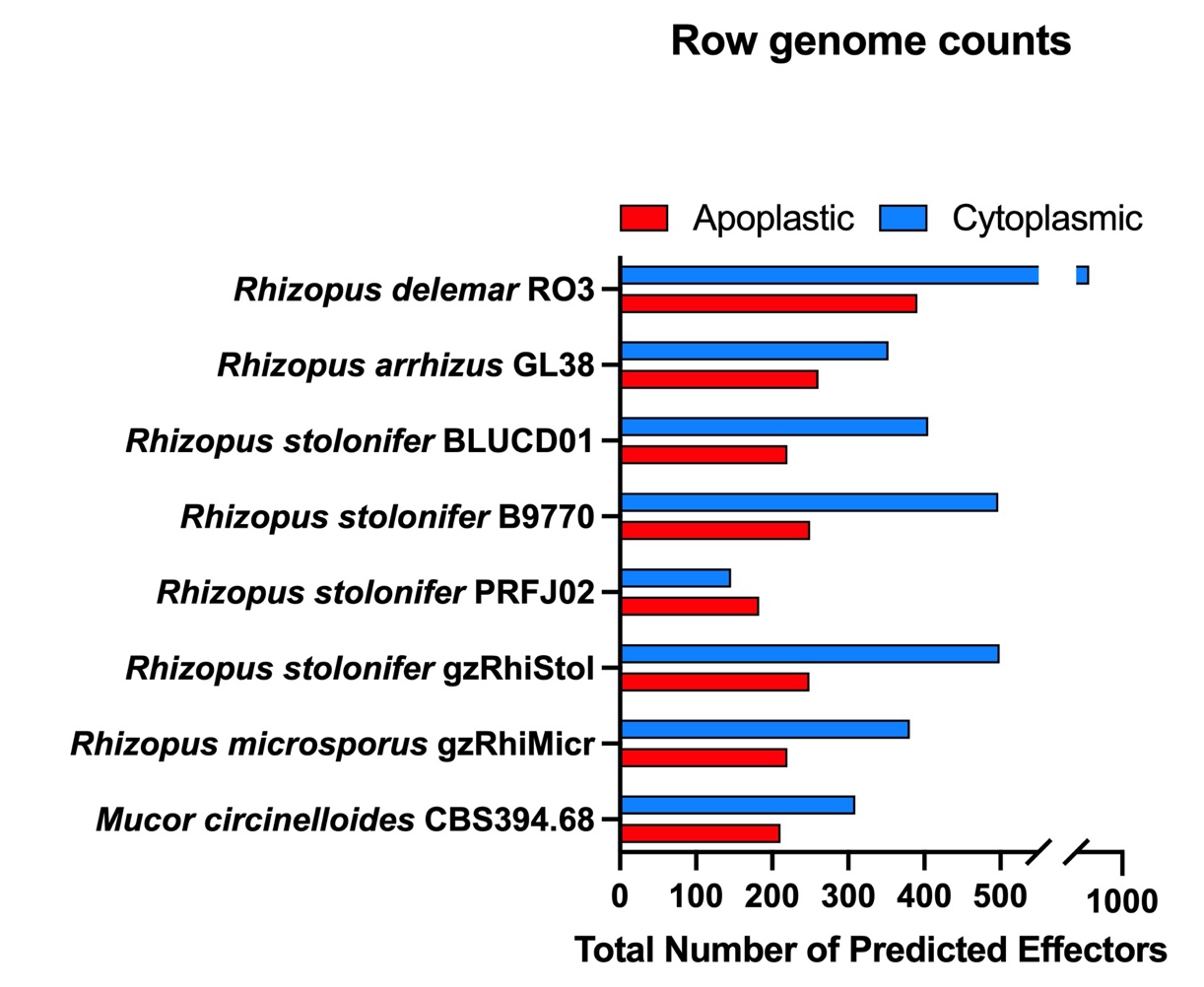


**Supplementary Figure S10.** Predicted number of apoplastic and cytoplasmic candidate effector genes in the Rhizopus genomes.

**
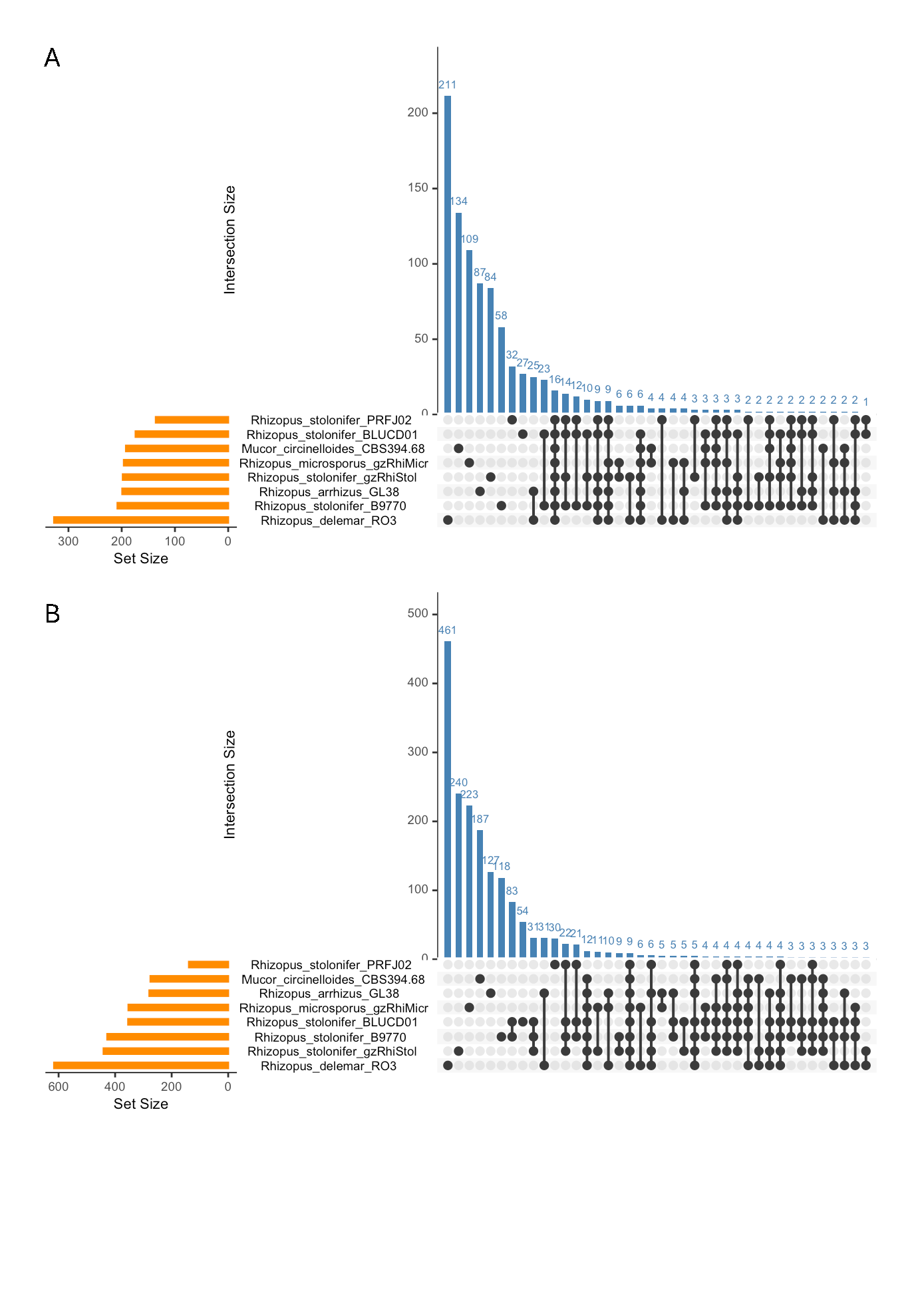
**

**Supplementary Figure S11.** UpSet plots illustrating shared and unique gene clusters among the Rhizopus genomes. (**A**) apoplastic and (**B**) cytoplasmic effectors were visualised across individual genomes and pairwise genome comparisons.

**Supplementary Table S1**. BUSCO Assessment on the Rhizopus genome assemblies. * Raw data is available in NCBI Bioproject (PRJNA1082801). Assembly is available at https://zenodo.org/records/7630319. Bold highlight indicates a BUSCO score over 95% and genome assemblies that were used in this comparative analysis.

| Assembly | **RhiStoB9770-1.0** | **Rstolonifer_PRFJ02_1.0** | **BLUCD01v1*** | **gzRhiStol1** | Rstol_CA | ASM4080714v1 | ASM1176360v1 | **ASM1180103v2** | **Mcir_PS15m_1.0** | **gzRhiMicr1** | **RO3** |
| --- | --- | --- | --- | --- | --- | --- | --- | --- | --- | --- | --- |
| Strain | B9770-1.0 | PRFJ02 | BLUCD01 | gzRhiStol | LSU 92-RS-03 | PG92-21 | GL27 | GL38 | CBS 394.68 | gzRhiMicr | RA 99-880 |
| Species | *R. stolonifer* | *R. stolonifer* | *R. stolonifer* | *R. stolonifer* | *R. stolonifer* | *R. stolonifer* | *R. stolonifer* | *R. arrhizus* | *M. circinelloides* | *R. microsporus* | *R. delemar* |
| Host | Contaminated product | Passion Fruit | Tomato | Culture (host not known) | Sweet potato | Human feces | Environment | Unknown | thawing beef | Culture | unknown |
| NCBI accession | GCA_000697035.1 | JBSRNQ000000000 | PRJNA1082801 | GCA_977110985.1 | GCA_003325415.1 | GCA_040807145.1 | GCA_011763605.1 | GCA_011801035.2 | GCA_051295215.1 | GCA_977110955.1 | GCF_000149305.1 |
| Assembly size | 38025998 | 48208381 | 45023700 | 41248464 | 29727802 | 18181348 | 38612125 | 42211743 | 37441900 | 28452767 | 46148878 |
| No. scaffold/contigs | 5567/5567 | 18/20 | 51/99 | 32/32 | 8064/10940 | 16636/16648 | 27595/29345 | 13326/14177 | 14/14 | 22/22 | 83/391 |
| Scaffold N50 | 23 Kb | 4 Mb | 2 Mb | 4 Mb | 4 Kb | 4 Kb | 3 Kb | 15 KB / 14 KB | 3 MB / 3 MB | 4 MB | 3 MB / 303 KB |
| BUSCO |  |  |  |  |  |  |  |  |  |  |  |
| **Complete Total** | **97.2% (1569)** | **98.9% (1596)** | **98.9% (1597)** | **98.7% (1593)** | 78.0% (1259) | 23.8% (384) | 81.7% (1319) | **98.2% (1585)** | **99.3% (1603)** | **99.3% 1602** | **99.0% (1598)** |
| Complete and single-copy | 91.2% (1472) | 92.8% (1497) | 92.7% (1496) | 92.9% (1500) | 54.6% (882) | 23.2% (375) | 78.6% (1269) | 88.2% (1424) | 99.3% (1603) | 99% (1598) | 88.9% (1435) |
| Complete and duplicated | 6.0 % (97) | 6.1% (99) | 6.3% (101) | 5.8% (93) | 23.4% (377) | 0.6% (9) | 3.1% (50) | 10.0% (161) | 0% (0) | 0.2% (4) | 10.1% (163) |
| Fragmented | 1.4 % (23) | 0.6% (10) | 0.6% (9) | 0.6% (9) | 6.8% (110) | 28.4% (458) | 12.3% (119) | 1.3% (21) | 0.2% (4) | 0.3% (5) | 0.6% (9) |
| Missing | 1.4 % (22) | 0.5% (8) | 0.5% (8) | 0.7% (12) | 15.2% (245) | 47.8% (772) | 5.9% (96) | 0.5% (8) | 0.4% (7) | 0.4% (7) | 0.4% (7) |
| Total | 1614 | 1614 | 1614 | 1614 | 1614 | 1614 | 1614 | 1614 | 1614 | 1614 | 1614 |

**Supplementary Table S2:** Summary of the joining and splitting of contigs for each scaffolding tool/pipeline.

| PRFJ02 contig assembly | Chromosome from original HiC scaffold assembly | YAHS scaffold assembly | SALSA2 scaffold assembly |
| --- | --- | --- | --- |
| 2 contigs, 2 telomeres | Joined 🡪 chr1 | Joined 🡪 T2T | Didn’t join (2 scaffolds, 2 telomeres) |
| 4 contigs, 1 telomere | Joined 3 contigs 🡪 chr2 (not T2T) | Joined 2 contigs (3 scaffolds, 1 telomere) | split (5 scaffolds, 1 telomeres) |
| 1 contig, 2 telomeres | chr3 | Split (2 scaffolds, 2 telomeres) | Split (2 scaffolds, 2 telomeres) |
| 1 contig, 2 telomeres | chr4 | T2T | T2T |
| 1 contig, 2 telomeres | chr5 | Split (2 scaffolds, 2 telomeres) | T2T |
| 1 contig, 2 telomeres | chr6 | T2T | Split (2 scaffolds, 2 telomeres) |
| 1 contig, 2 telomeres | chr7 | T2T | T2T |
| 1 contig, 2 telomeres | chr8 | T2T | T2T |
| 1 contig, 2 telomeres | chr9 | T2T | T2T |
| 1 contig, 2 telomeres | chr10 | Split (2 scaffolds, 2 telomeres) | T2T |
| 1 contig, 2 telomeres | chr11 | T2T | T2T |

**Supplementary Table S3.** Summary of QC scores for the Hi-C scaffolded PRFJ02 assemblies.

|  | **Original contig assembly** | **Original HiC scaffold assembly** | **Chromap + YaHS pipeline** | **Chromap + SALSA2 pipeline** |
| --- | --- | --- | --- | --- |
| **No. scaffolds** | 21 | 18 | 22 | 23 |
| **Total length bp** | 48208123 | 47866798 | 48208323 | 48209623 |
| **Largest scaffold** | 6159681 | 6651178 | 6650994 | 6159681 |
| **# scaffolds (>= 50000 bp)** | 16 | 11 | 17 | 19 |
| **N50** | 4233866 | 4366636 | 3665085 | 3665085 |
| **L50** | 5 | 5 | 6 | 6 |
| **L90** | 10 | 10 | 12 | 12 |
| **GC%** | 35.99 | 35.98 | 35.99 | 35.99 |
| **Busco %** | 94.90% | 95% | 94.70% | 94.70% |

**Supplementary Table S4.** Identification of repetitive elements in the genome of *Rhizopus stolonifer* PRFJ02 using RepeatMasker.

| **Category** | **Subcategory** | **No. of elements** | **Length occupied (bp)** | **% sequence** |
| --- | --- | --- | --- | --- |
| Retroelements |  | 8214 | 1608937 | 3.34 |
| SINEs: |  | 186 | 9670 | 0.02 |
|  | Penelope | 332 | 24174 | 0.05 |
| LINEs: |  | 2797 | 192494 | 0.4 |
|  | CRE/SLACS | 10 | 625 | 0 |
|  | L2/CR1/Rex | 436 | 22567 | 0.05 |
|  | R1/LOA/Jockey | 261 | 28337 | 0.06 |
|  | R2/R4/NeSL | 132 | 5688 | 0.01 |
|  | RTE/Bov-B | 125 | 6383 | 0.01 |
|  | L1/CIN4 | 925 | 59443 | 0.12 |
| LTR elements: |  | 5231 | 1406773 | 2.92 |
|  | BEL/Pao | 142 | 12103 | 0.03 |
|  | Ty1/Copia | 596 | 37837 | 0.08 |
|  | Gypsy/DIRS1 | 3720 | 1314958 | 2.73 |
|  | Retroviral | 563 | 26178 | 0.05 |
| DNA transposons | | 7429 | 1187909 | 2.46 |
|  | hobo-Activator | 1063 | 51966 | 0.11 |
|  | Tc1-IS630-Pogo | 1642 | 465853 | 0.97 |
|  | En-Spm | 0 | 0 | 0 |
|  | MULE-MuDR | 493 | 32345 | 0.07 |
|  | PiggyBac | 48 | 2680 | 0.01 |
|  | Tourist/Harbinger | 317 | 56306 | 0.12 |
|  | Other (Mirage, P-element, Transib) | 120 | 4819 | 0.01 |
| Rolling-circles |  | 484 | 77990 | 0.16 |
| Unclassified: |  | 23127 | 16223777 | 33.65 |
| Total interspersed repeats: | |  | 19020623 | 39.46 |
| Small RNA: |  | 0 | 0 | 0 |
| Satellites: |  | 193 | 13316 | 0.03 |
| Simple repeats: |  | 0 | 0 | 0 |
| Low complexity |  | 0 | 0 | 0 |

**Supplementary Table S5.** rRNA and tRNA genes identified in the genome of PRFJ02.

| **Type** | **Copy** | **Average_length(bp)** | **Total_length(bp)** | **% of genome** |
| --- | --- | --- | --- | --- |
| 5S rRNA | 23 | 109.6 | 2521 | 0.005 |
| 5.8S rRNA | 19 | 150.0 | 2850 | 0.006 |
| 18S rRNA | 22 | 1636.8 | 36009 | 0.075 |
| 28S rRNA | 23 | 3464.7 | 79688 | 0.165 |
| tRNA | 398 | 74.6 | 29695 | 0.062 |

**Supplementary Table S6.** Clustering of *Rhizopus stolonifer* isolate PRFJ02 proteins based on the functional classification of KOG. The three most abundant categories are highlighted in bold.

| **KOG categories** | **categories function** | **ORF number** |
| --- | --- | --- |
| A | RNA processing and modification | 344 |
| B | Chromatin structure and dynamics | 113 |
| C | Energy production and conversion | 367 |
| D | Cell cycle control, cell division, chromosome partitioning | 154 |
| E | Amino acid transport and metabolism | 310 |
| F | Nucleotide transport and metabolism | 104 |
| G | Carbohydrate transport and metabolism | 459 |
| H | Coenzyme transport and metabolism | 101 |
| I | Lipid transport and metabolism | 348 |
| **J** | **Translation, ribosomal structure and biogenesis** | **611** |
| K | Transcription | 533 |
| L | Replication, recombination and repair | 217 |
| M | Cell wall/membrane/envelope biogenesis | 86 |
| N | Cell motility | 4 |
| **O** | **Posttranslational modification, protein turnover, chaperones** | **612** |
| P | Inorganic ion transport and metabolism | 198 |
| Q | Secondary metabolites biosynthesis, transport and catabolism | 187 |
| R | General function prediction only | 0 |
| S | Function unknown | 1440 |
| **T** | **Signal transduction mechanisms** | **625** |
| U | Intracellular trafficking, secretion, and vesicular transport | 468 |
| V | Defense mechanisms | 42 |
| W | Extracellular structures | 4 |
| Y | Nuclear structure | 4 |
| Z | Cytoskeleton | 175 |

**Supplementary Table S7.** Overall results of the protein orthologous clustering analysis using Orthovenn3 and *R. stolonifer* isolates PRFJ02, gzRhiStol, B9770, BLUCD01, and *R. microsporus* isolate gzRhiMicr, *R. delemar RA* isolate RO3, *R. arrhizus* isolate GL38, and *Mucor circinelloides* isolate CBS 394.68. Overlaps refer to the number of orthologous gene clusters in the single intersection pattern with the most clusters, as reported by OrthoVenn3. This is not equivalent to the total number of clusters shared between genomes. Single-copy clusters are clusters containing exactly one gene per isolate. Non-overlapping clusters correspond to clusters unique to a single isolate.

| Overlaps | 217 |
| --- | --- |
| All clusters | 14913 |
| Single-copy clusters | 3055 |
| All proteins | 112001 |
| All singletons | 13053 |
| Percentage of singletons | 11.65% |

**Supplementary Table S8.** Overall results of the protein orthologous clustering analysis using Orthovenn3 on the four *Rhizopus stolonifer* strains, PRFJ02, gzRhiStol, B9770 and BLUCD01. Overlaps refer to the number of orthologous gene clusters in the single intersection pattern with the most clusters, as reported by OrthoVenn3. This is not equivalent to the total number of clusters shared between genomes. Single-copy clusters are clusters containing exactly one gene per isolate. Non-overlapping clusters correspond to clusters unique to a single isolate.

| Overlaps | 15 |
| --- | --- |
| All clusters | 12308 |
| Single-copy clusters | 7903 |
| All proteins | 53609 |
| All singletons | 4702 |
| Percentage of singletons | 8.77% |
